# Inactivation of the CIC-DUX4 oncogene through P300/CBP inhibition, a therapeutic approach for CIC-DUX4 sarcoma

**DOI:** 10.1038/s41389-021-00357-4

**Published:** 2021-10-12

**Authors:** Darko Bosnakovski, Elizabeth T. Ener, Mark S. Cooper, Micah D. Gearhart, Kevin A. Knights, Natalie C. Xu, Christian A. Palumbo, Erik A. Toso, Graham P. Marsh, Hannah J. Maple, Michael Kyba

**Affiliations:** 1 Lillehei Heart Institute, Minneapolis, USA; 2 Department of Pediatrics, University of Minnesota, Minneapolis, MN 55455 USA; 3 Faculty of Medical Sciences, University Goce Delchev - Shtip, Shtip, 2000 Republic of North Macedonia; 4 Bio-Techne (Tocris), The Watkins Building, Atlantic Road, Avonmouth, Bristol, UK; 5 Department of Genetics, Cell Biology and Development, University of Minnesota, Minneapolis, MN 55455 USA

**Keywords:** Oncogenes, Cancer genetics

## Abstract

CIC-DUX4 sarcoma (CDS) is a highly aggressive and metastatic small round type of predominantly pediatric sarcoma driven by a fusion oncoprotein comprising the transcriptional repressor Capicua (CIC) fused to the C-terminal transcriptional activation domain of DUX4. CDS rapidly develops resistance to chemotherapy, thus novel specific therapies are greatly needed. We demonstrate that CIC-DUX4 requires P300/CBP to induce histone H3 acetylation, activate its targets, and drive oncogenesis. We describe the synthetic route to a selective and highly potent P300/CBP inhibitor named iP300w and related stereoisomers, and find that iP300w efficiently suppresses CIC-DUX4 transcriptional activity and reverses CIC-DUX4 induced acetylation. iP300w is active at 100-fold lower concentrations than related stereoisomers or A-485. At low doses, iP300w shows specificity to CDS cancer cell lines, rapidly inducing cell cycle arrest and preventing growth of established CDS xenograft tumors when delivered in vivo. The effectiveness of iP300w to inactivate CIC-DUX4 highlights a promising therapeutic opportunity for CDS.

## Introduction

A newly recognized commonly pediatric subtype of undifferentiated round cell sarcoma that is driven by fusion between the cell cycle regulator and transcriptional repressor, *capicua (CIC)*, and the transcriptional activator, *DUX4*, has been identified based on its histology, clinical differences, and aggressiveness [1–8]. CIC-DUX4 sarcoma (CDS) occurs predominantly in children and young adults and usually develops in somatic soft tissues [4, 5, 9, 10]. Patients with CDS show an aggressive clinical course with a high metastatic rate and quickly develop resistance to chemotherapy. The median survival is less than 2 years [6, 9, 11]. CDS at the molecular level is poorly understood and so far, all of the treatment efforts have poor and unsatisfactory outcomes.

The *CIC-DUX4* fusion is the product of a translocation between the *Capicua Transcriptional Repressor* (*CIC*) on chromosome 19 and *DUX4* on chromosome 4 (4;19)(q35;q13), or on rare occasions with *DUX4* from chromosome 10 (10;19)(q26;q13) [1, 7]. *DUX4* is the double homeobox transcription factor gene embedded in each D4Z4 macrosatellite repeat located at both 4q35 and 10q26.3 [12, 13]. The CIC–DUX4 oncoprotein contains the majority of the N-terminal part of CIC, which encompasses its DNA binding domain, and a small C-terminal part of DUX4 that has strong transcriptional activation properties [8, 14, 15]. The acquisition of the DUX4 C-terminus transforms CIC from a transcriptional repressor into an activator [8]. Crucial cell cycle genes, as well as genes involved in driving metastasis previously repressed by CIC, are expressed at high levels, driving uncontrolled cell division and malignant transformation [8, 16, 17].

DUX4 is known for its involvment in early embryogenesis [18–20] and facioscapulohumeral muscular dystrophy (FSHD) [21–23]. We have shown that the C-terminus of DUX4 recruits P300/CBP and induces both local histone (H3) acetylation and total nuclear histone H3 hyperacetylation [15]. Deletion of the C-terminus of *DUX4* eliminates induced H3 acetylation and target gene expression [14, 15, 24]. Similar effects were achieved by specific P300 inhibition in cells expressing DUX4 [25].

Because CIC-DUX4 acquires the p300-interacting activation domain of DUX4, we hypothesized that its transcriptional activation potential, and thus the oncogenicity of CIC-DUX4, would require P300 or its homolog, CBP. Recently, a new class of histone acetyltransferase inhibitor with high selectivity for P300 and CBP has been described, and two compounds, A-485, and iP300w, have been shown to inhibit P300 and CBP both in vitro and in vivo [25, 26], with iP300w having the ability to reverse gene expression changes caused by DUX4. If this hypothesis is correct, iP300w should potently counteract the CIC-DUX4 gene expression program and might be a particularly effective therapy for CDS. In this study, we confirm this hypothesis, demonstrating that CIC-DUX4 requires P300/CBP for its activity. We also demonstrate that CIC-DUX4 induces a global increase in H3 acetylation, like DUX4, which is reversible with iP300w treatment. We find that CIC-DUX4 activity is potently blocked by iP300w, and that this compound has potent activity against CDS cell lines in vitro, and in an in vivo cancer xenograft assay.

## Results

### CIC-DUX4 acts through P300/CBP

**Fig. 1:**
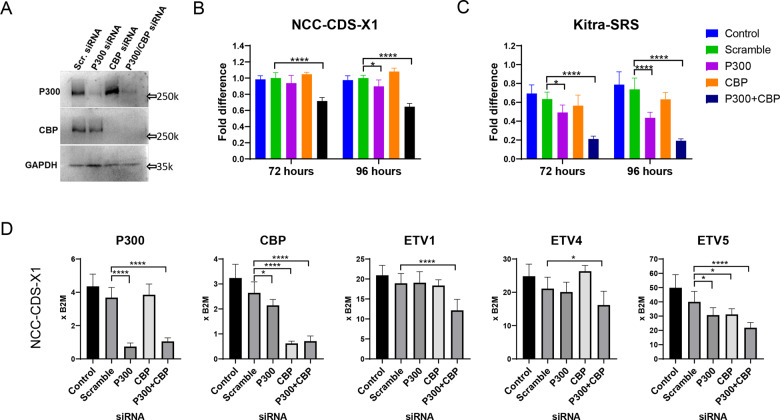
P300/CBP is required for CIC-DUX4 activity. **A** Western blots for P300 and CBP in NCC-CDS-X1 cells after 96 h transfection with siRNAs for P300 and CBP. **B** ATP assay for cell viability of NCC-CDS-X1 at 72 and 96 h of post-transfection with P300 and/or CBP siRNA. Non-treated cells and cells transfected with non-targeting siRNA (scramble) were used as negative controls. Data are presented as mean ± SEM; *p < 0.05, ***p < 0.001 by one-way ANOVA. Results are presented as relative expression to control (*n* = 4). **C** ATP viability assay in the Kitra-SRS cell line in the same experimental conditions as explained in “**B**”. Data are presented as mean ± SEM; ****p < 0.0001, by two-way ANOVA. Results are presented as expression relative to control (*n* = 4). **D** RT-qPCR for P300, CBP, and CIC-DUX4 target genes in NCC-CDS-X1. Analyses were performed 48 h post-transfection with P300 and/or CBP siRNA. Data are presented as mean ± SEM; *p < 0.05, **p < 0.01, ***p < 0.001 by one-way ANOVA. Results are presented as expression relative to *B2M* (*n* = 3).

We previously showed that the C-terminus of DUX4 interacts with P300 and induces both DUX4 target locus-specific acetylation as well as a global elevation of acetylation on H3K18 and H3K27 [15, 25]. Thus, it is likely that CIC-DUX4 also requires P300/CBP for activation of its targets. To test this hypothesis, we knocked down P300 and CBP in two cell lines, NCC-CDS-X1 and Kitra-SRS, derived from independent CDS tumors [27, 28] and evaluated cell viability and CIC-DUX4 target gene expression in these cells. The efficiency of the knockdown was confirmed by western blot (Fig. 1A). Co-transfection with P300 and CBP siRNA resulted in significant reductions of cell viability in both cell lines at 72 and 96 h of post-transfection (Fig. 1B, C). Notably, a slight but still significant decrease in cell proliferation was detected in the cells in which only P300 was targeted. The knockdown also resulted in reductions of the key CIC-DUX4 target genes, *ETV1*, *4*, and *5* (Fig. 1D and Supplementary Fig. 1). *ETV4* is a well-established CIC-DUX4 target and cancer metastatic driver that has been used to distinguish CDS from the other Ewing sarcomas [8, 10, 16, 29]. Thus, we conclude that CIC-DUX4 transcriptional activity and with that, CDS proliferation and/or survival, depends on P300/CBP.

### Synthesis and stereochemical evaluation of iP300w, a potent P300/CBP inhibitor

**Fig. 2:**
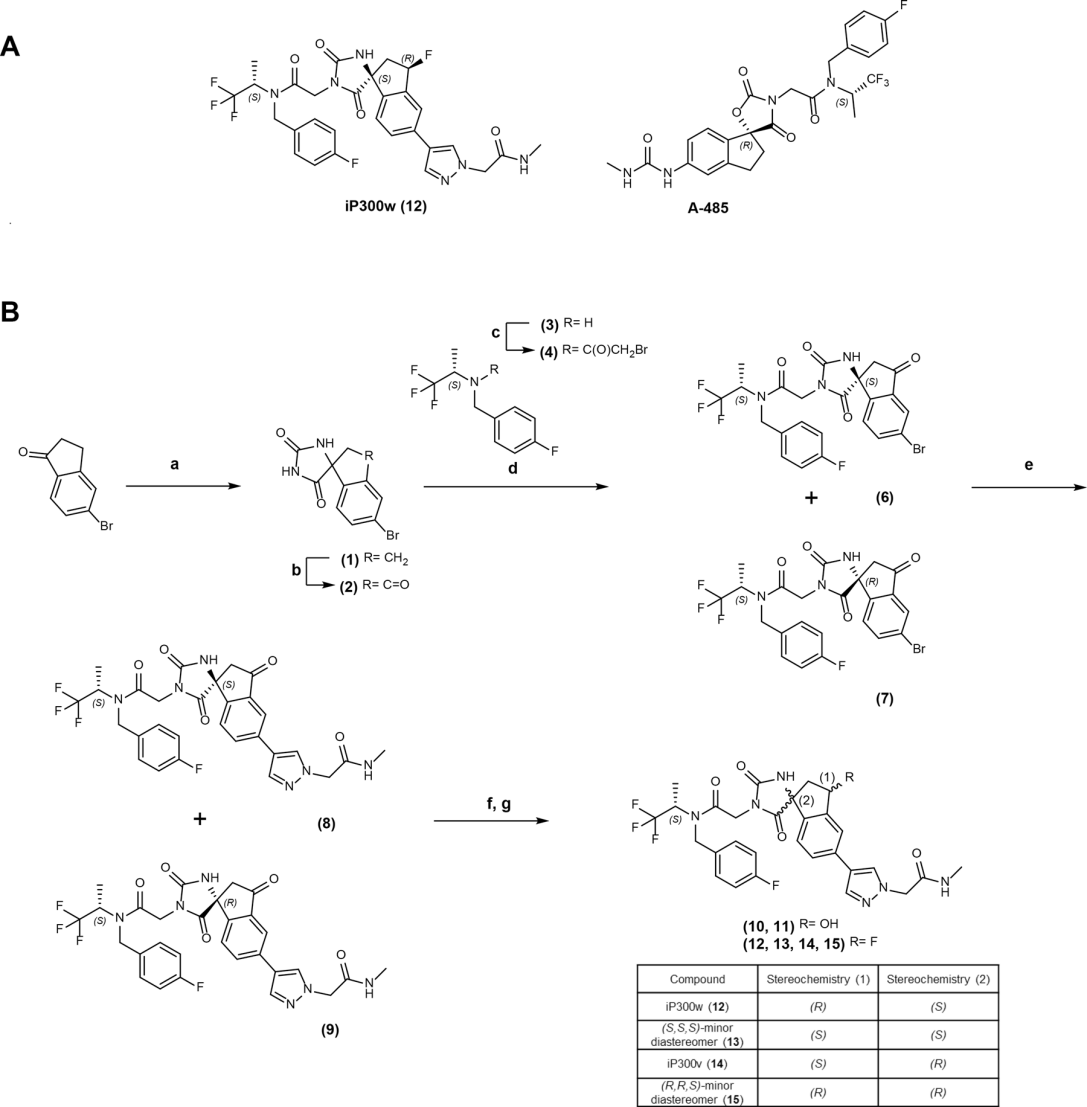
Synthesis and structure of iP300w and stereoisomers. **A** Structures of iP300w and A-485. **B** Synthesis pathway, reagents and conditions: a KCN, (NH4)2CO3, EtOH, H_2_O, 70 °C; b sodium 2-iodobenzenesulfonic acid, (CH_3_CH_2_CH_2_CH_2_)4N(HSO_4_), Oxone, MeCN, 65 °C; **c** bromoacetyl bromide, DCM, RT; **d** K2CO3, DMF, 5 °C, followed by preparative chromatography; **e** N-methyl-2-(4-(4,4,5,5-tetramethyl-1,3,2-dioxaborolan-2-yl)-1H-pyrazol-1-yl)acetamide, Pd(dppf)Cl2.CH2Cl2, K2CO3, 1,4-dioxane, H_2_O, 95 °C; **f** NaBH4, THF, MeOH, 0 °C; **g** DAST, DCM, −70 °C.

The first potent and highly selective inhibitor of the HAT domain of P300 and CBP to be reported was A-485 (Fig. 2A) [26], a competitive binder for the acetyl co-A site of P300. Several structurally related compounds from the same series have recently been described to have P300 inhibitory activity [25, 30–32]. One of these, iP300, was shown to have activity residing in a single diastereomer with undefined stereochemistry, named ‘iP300w’ (Fig. 2A) [25]. A second undefined diastereomer (Comp 13) exhibited substantially reduced activity [25]. Given the structural similarities with A-485, iP300w might reasonably be assumed to share the same stereochemical configuration at both the central spirocyclic core and adjacent to the terminal trifluoromethyl moiety (*(R)* and *(S)* respectively in A-485). Contrary to this assumption, the stereochemical assignment from the original patent (WO2016044770) provides the spirocyclic core of iP300w as *(S)*, and it has been further illustrated that the preferred stereochemistry at this center for such spiro-hydantoin compounds is surprisingly opposite to the comparable spiro-oxazolidinediones [30, 32].

We synthesized and isolated four diastereomers of iP300 according to the route shown (Fig. 2B). From commercially available 5-bromoindan-1-one, a Bucherer-Bergs reaction facilitated elaboration to the hydantoin (**1**), with subsequent oxone-mediated benzylic oxidation affording (**2**). Alkylation with enantiomerically pure bromide (**4**) gave rise to a mixture of (**6**) and (**7**), which were separated and independently advanced to the end of the synthesis. Suzuki-Miyaura coupling afforded (**8**) and (**9**), with the final stereocenter being installed by a two-stage reduction and fluorination sequence.

Compound (**12**) co-eluted by HPLC with an authentic sample of iP300w from the original study (Bosnakovski et al. [25], while compound (**14**) co-eluted with an authentic sample of iP300v. The absolute stereochemistry of (**14**) (iP300v) was determined by single crystal X-ray diffraction as being *(R)* at the spirocycle and *(S)* at the fluoro-substituent (Supplementary Fig. 2A). Furthermore, analysis of precursor (**6**) (Supplementary Fig. 2B) by single crystal X-ray diffraction revealed the spirocyclic core being in the *(S)* configuration, unequivocally confirming the absolute stereochemical arrangement of the active diastereomer (**12**) (iP300w).

### iP300w and related compounds decrease CIC-DUX4 sarcoma cell viability

**Fig. 3:**
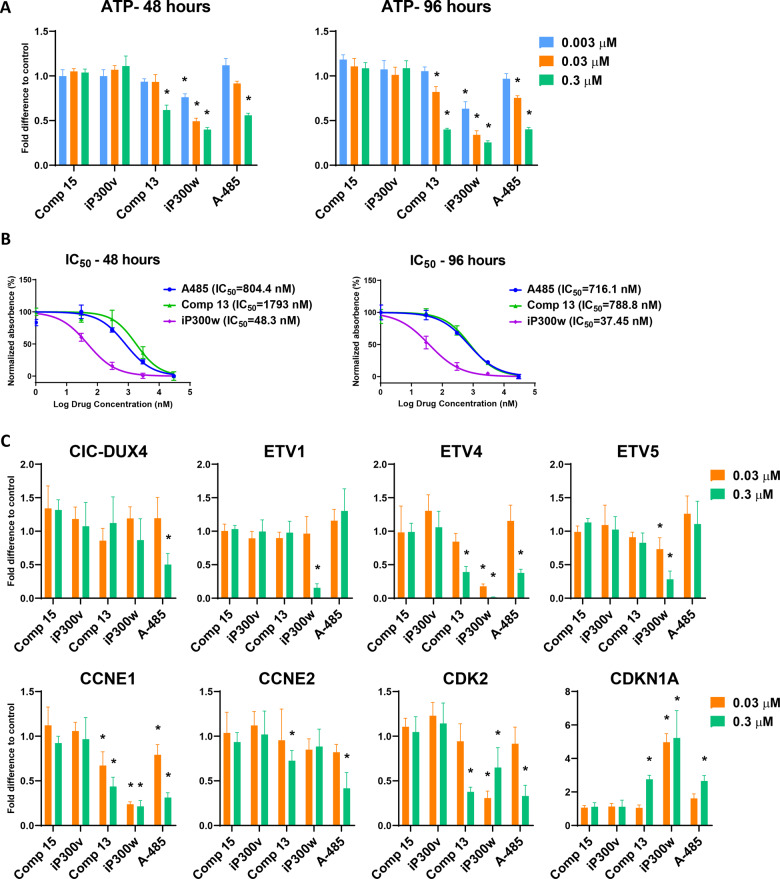
Effect of iP300w stereoisomers on CIC-DUX4 sarcoma cell line viability. **A** ATP viability assays at 48 and 96 h of treatment of NCC-CDS-X1 cells with different concentrations of iP300w stereoisomers and A-485. **B** IC_50_ values of A-485, Comp 13 and iP300w in NCC-CDS-X1 cells at 48 and 98 h of treatment. **C** RT-qPCR for CIC-DUX4 target genes in NCC-CDS-X1 cells after 24 h treatment with 0.03 and 0.3 µM iP300w stereoisomers and A-485. Data is presented as mean ± SEM; *p < 0.05, by two-way ANOVA. Results are presented as expression relative to control (*n* = 3).

We evaluated the activity of the various iP300w stereoisomers against NCC-CDS-X1 cells. Cultures were treated with serial dilutions, ranging from 0.003 to 3.0 µM, for up to 96 h. In cells treated with iP300w, a significant decrease in cell viability was evident at the lowest concentration (0.003 µM) at 48 and 96 h (Fig. 3A). The effect was dose-dependent and time-dependent. In addition to iP300w, two compounds, stereoisomer Comp 13, and related compound A-485, also showed activity, but at the early time point (48 h) only at the highest concentrations. We calculated IC_50_ values and concluded that Comp 13 and A-485 are approximately 20-fold less potent than iP300w. (Fig. 3B and Supplementary Fig. 3).

Next, we analyzed the effect of the stereoisomers on CIC-DUX4 and its target genes in cells cultured with the compounds for 24 h. Except for A-485 at the highest concentration (0.3 µM), none of the compounds significantly affected CIC-DUX4 expression (Fig. 3C). We detected significant downregulation of all three direct targets, ETV1, 4, and 5 in the group treated with iP300w (Fig. 3C). Comp 13 and A-485 at the highest concentration also had a notable effect on ETV4. Cell cycle genes, including a known CIC-DUX4 target CCNE-CDK2 complex [16], were also affected by iP300w (Fig. 3C). Taken together, based on the cell viability assay and the gene expression analyses, we identified iP300w as the most potent inhibitor of CIC-DUX4.

### iP300w inhibits CIC-DUX4 sarcoma cell line proliferation

**Fig. 4:**
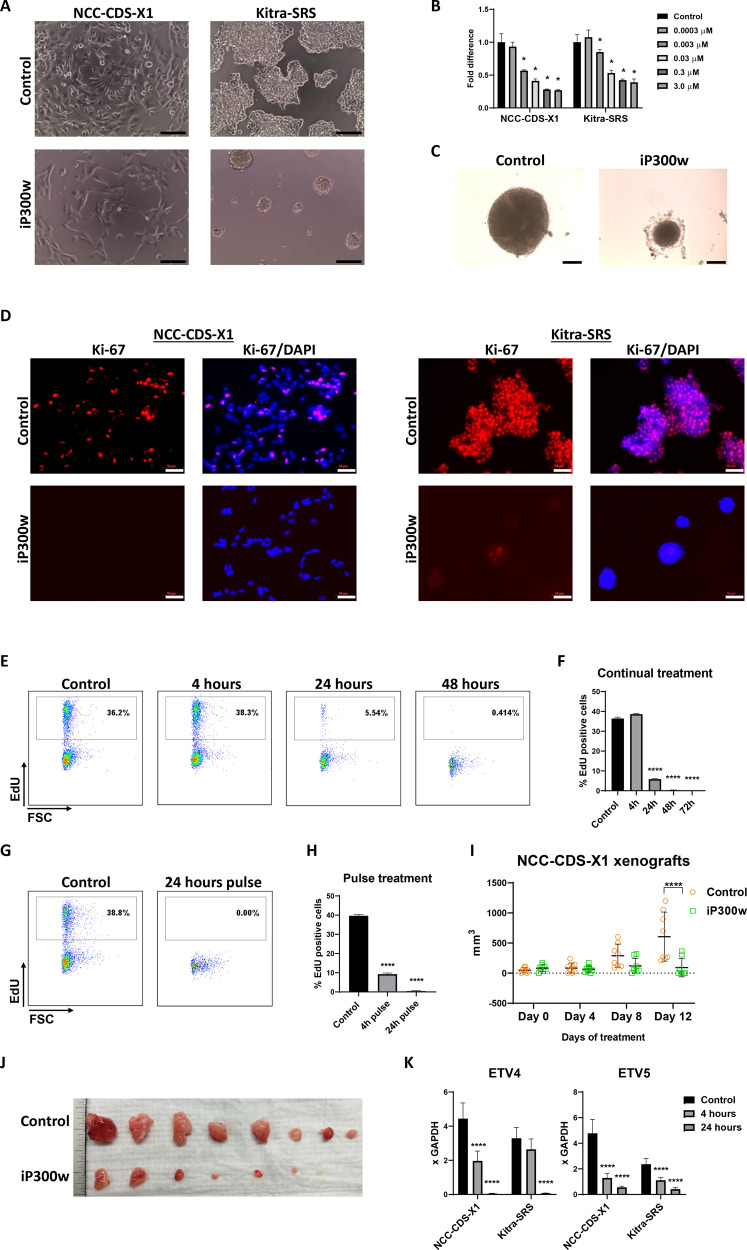
iP300w suppresses CIC-DUX4 sarcoma cell proliferation in vivo and in vitro. **A** Morphology of NCC-CDS-X1 and Kitra-SRS cell cultures at 48 h of treatment with iP300w (0.3 µM). Scale bar, 50 µm. **B** ATP assay for cell viability at 48 h of treatment with a serial dilution of iP300w. **C** NCC-CDS-X1 spheroid morphology after 4 days of treatment with iP300w (0.3 µM). Scale bar, 50 µm. **D** Immunofluorescence for Ki-67 in NCC-CDS-X1 and Kitra-SRS cells treated for 24 h with iP300w (0.3 µM). Scale bar, 50 µm. **E** Representative FACS analyses for EdU incorporation in NCC-CDS-X1 cells. Cells were treated with 0.3 µM iP300w for 4, 24, and 48 h. In the last 4 h of treatment proliferating cells were labeled with EdU (10 µM). **F** Summary of the FACS analyses presented in “**E**”. Data are presented as mean ± SEM; *p < 0.05, by one-way ANOVA. Results are presented as expression relative to control (*n* = 3). **G** Representative FACS analyses for EdU incorporation in NCC-CDS-X1 cells after a pulse of iP300w (0.3 µM). Cells were incubated for different periods with iP300w (0.3 µM) and analyzed by FACS 24 h later. In the last 4 h of the experiment, the cells were incubated with EdU. **H** Summary of FACS analyses presented in “**G**”. **I** Size of the NCC-CDS-X1 xenograft tumors in control and treated mice over 12 days. Mice (*n* = 8) were treated 1.4 mg/kg iP300w twice daily. **J** Gross morphology of dissected tumors at the endpoint of the experiment (day 12) presented in “**I**”. **K** RT-qPCR for ETV4 and ETV5 in NCC-CDS-X1 and Kitra-SRS cell lines after 4 and 24 h of treatment with 0.3 µM iP300w. Data are presented as mean ± SEM; *p < 0.05, **p < 0.01, ***p < 0.001 by one-way ANOVA. Results are presented as expression relative to *GAPDH* (*n* = 3).

To more rigorously evaluate the consequences of iP300w treatment of CDS cells, we monitored its effects on both monolayer as well as 3D spheroid cultures and in both CDS cell lines. Inhibition of proliferation was morphologically apparent as early as 24 h after iP300w treatment (not shown) and progressed with time (Fig. 4A). Using the cell viability assay, we determined that 0.003 µM was the lowest effective concentration on day 2 in both lines (Fig. 4B). The dose- and time-responsive inhibitory effect of iP300w on CDS cell spheroid formation and growth was also evident (Fig. 4C and Supplementary Fig. 4). Two independent approaches, Ki-67 staining and EdU incorporation revealed that iP300w treatment arrested proliferation of most cells. An almost complete absence of Ki-67 staining was observed in both NCC-CDS-X1 and Kitra-SRS cells at 24 (Fig. 4D) hours, and progressive reductions were obtained with EdU labeling until virtually no cells were labeled at 48 h (Fig. 4E, F). We also tested CDS cells for the effects of pulses of iP300w exposure. Interestingly, a 4 h pulse significantly reduced proliferation rate measured 20 h later (Fig. 4G, H). The effect was even more dramatic in the cells treated for 24 h, as they were not able to restore the cell cycle 48 h after the pulse (Fig. 4G, H).

We treated pancreatic and colorectal cancer cell lines with iP300w to further distinguish its specificity to CDS from general cytotoxicity. p300/CBP have been correlated with the invasive and migratory properties of pancreatic and colorectal cancers [33, 34]. Notably, we did not observe any significant effect on cell viability in the tested cell lines (Supplementary Fig. 5A, B). However, we did notice a mild suppression of proliferation of iP300w in LHCN-M2 cells at a concentration of 0.3 µM or higher after 3 days of treatment (Supplementary Fig. 5C).

We evaluated the effectiveness of iP300w to suppress CDS tumor growth in vivo. NCC-CDS-X1 cells were used to generate subcutaneous xenograft tumors in immunodeficient NSG mice (*n* = 8). When tumors were palpable (day 9) iP300w injections (1.4 mg/kg b.i.d.) were initiated and the progression of tumor growth was monitored. While tumor growth was evident in the control mice, growth was halted (5/8) or significantly diminished (3/8) in the treated mice (Fig. 4I, J).

Finally, we confirmed that iP300w effectively suppresses transcription of CIC-DUX4 direct target genes ETV4 and ETV5 in both NCC-CDS-X1 and Kitra-SRS cell lines at 24 h of treatment (Fig. 4K).

### iP300w suppresses the CIC-DUX4 induced transcriptome

**Fig. 5:**
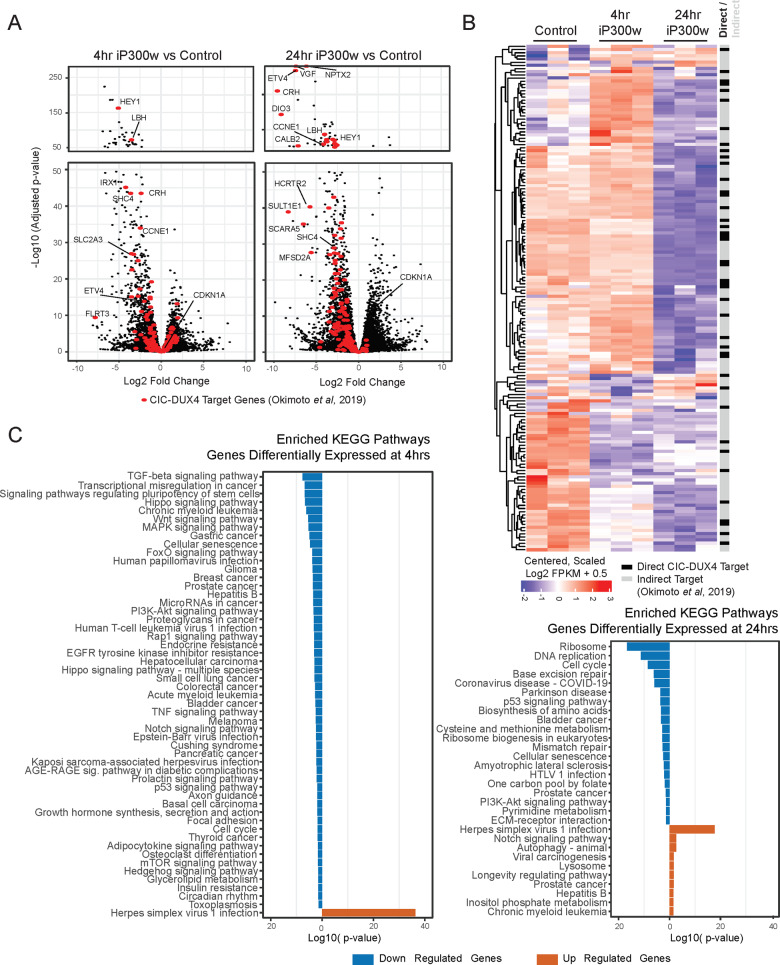
iP300w suppresses the CIC-DUX4 target transcriptome. **A** Scatter plots of −log10 fold change of the Benjamini–Hochberg adjusted *p*-value versus the observed log2 fold change in gene expression. The changes with 4 and 24 h of iP300w treatment are shown on the left and right, respectively. The *y*-axis in each plot has been expanded in the lower panels relative to the upper panels. Points colored in red correspond to the 165 transcripts upregulated by CIC-DUX4 as previously reported [16]. **B** Heatmap of the log2 padded FPKM values of CIC-DUX4 targets from Okimoto et al. [16] in NCC-CDS1-X1 cells upon treatment with iP300w. **C** Enriched KEGG pathways (hypergeometric test, Benjamini–Hochberg adjusted *p*-value < 0.05) of the differentially expressed genes at 4 h (left) and 24 h (lower right). The *x*-axis corresponds to the log10 adjusted *p*-value such that downregulated genes are shown on the negative axis (blue) and upregulated genes (orange) are shown on the positive axis in each plot.

We next investigated global transcriptional profiles of NCC-CDS-X1 cells treated with iP300w. RNA-seq performed after treatment with iP300w identified 1182 downregulated and 872 upregulated genes 4 h post-treatment and 2042 downregulated and 1658 upregulated genes 24 h post-treatment (2-fold or greater change in gene expression (log2FC > 1 or < −1), Benjamini–Hochberg adjusted *p*-value <0.05, mean counts across samples >10 and FPKM in the control or iP300w-treated sample >2.5). Among these differentially expressed genes, 170 and 244 were changed by more than 10-fold at 4 h and 24 h, respectively (Fig. 5A and Supplementary Table 1). As numerous genes are dependent on P300 acetyltransferase activity, we were particularly interested in genes that are known to be direct targets of CIC-DUX4. Using the set of 165 CIC-DUX4 targets identified by Okimoto et al. [16], we found numerous key transformation-regulatory genes, including *ETV1*, *ETV4*, and *CCNE1*, to be significantly downregulated within 4 h of iP300w treatment, and the vast majority of CIC-DUX4 targets to be downregulated by 24 h of iP300w treatment (Fig. 5B). This contrasts with the global background of gene expression changes, which were divided among upregulation and downregulation, and argues that iP300w has a predominately antagonistic effect on the genes induced by the oncogenic CIC-DUX4 transformation. Further supporting this notion is that one of the rare upregulated genes was p21 (*CDKN1A*, upregulated in the 24 h dataset).

Because iP300w treatment affected many genes not previously described as CIC-DUX4 targets, we performed enrichment analysis with Kyoto Encyclopedia of Genes and Genomes (KEGG) pathways with the upregulated and downregulated genes (Fig. 5C). Several pathways related to cancer, as well as signaling pathways frequently misregulated in cancer, were enriched among the genes downregulated at 4 h, including the TGF-beta, WNT, and MAPK signaling pathways. By 24 h the enriched pathways for downregulated genes also included ribosome biogenesis, DNA replication, and cell cycle consistent with previously observed inhibition of cell cycle. A similar set of pathways was enriched among the downregulated genes previously identified as CIC-DUX4 targets. We identified a set of 30 iP300w responsive genes among the CIC-DUX4 targets that contribute to these pathways including *ETV1*, *ETV4, ETV5*, *CCNE1*, and *TGFB3* as central to the transformation by CIC-DUX4 that can be reversed by inhibiting P300/CBP (Supplementary Fig. 6).

### CIC-DUX4 induces global H3 acetylation, which is reversed by iP300w

**Fig. 6:**
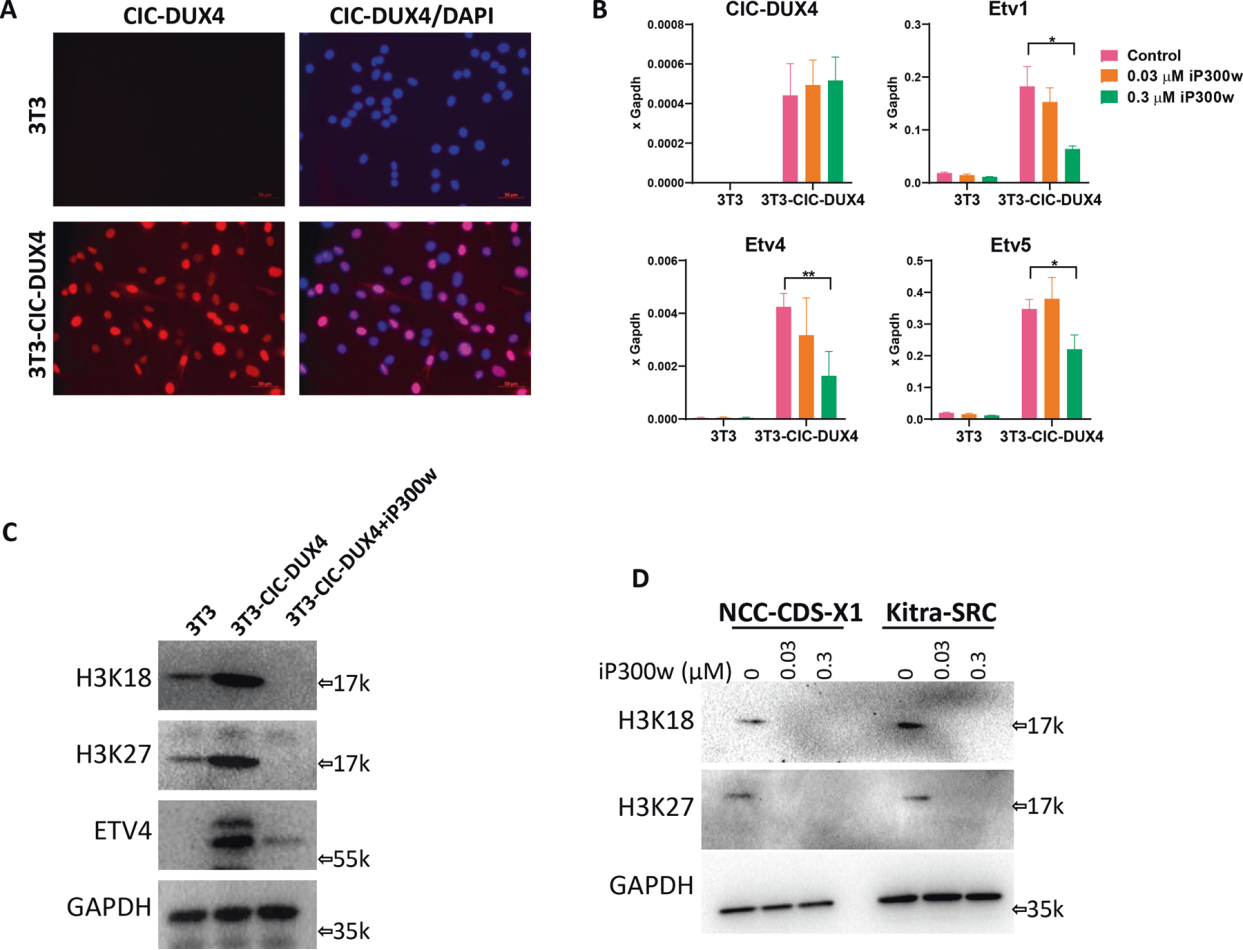
CIC-DUX4 induces H3 acetylation that is reversible by iP300w. **A** Immunostaining for CIC-DUX4 in the 3T3-CIC-DUX4 cell line. **B** RT-qPCR for CIC-DUX4 and its target genes in 3T3-CIC-DUX4 cells treated for 18 h with iP300w. **C** Western blots for ETV4 and acetylated H3K18 and H3K27 in 3T3-CIC-DUX4 cells treated for 18 h with iP300w. **D** Western blots for H3 acetylation markers in CIC-DUX4 sarcoma cell lines treated for 18 h with iP300w.

Finally, we have previously shown that DUX4 expression leads to a global increase of H3K18 and H3K27 acetylation, through a mechanism that is as yet undetermined, but dependent on P300 activity [25]. We wondered whether CIC-DUX4, which bears the P300 interaction domain of DUX4, would have this same activity. We therefore generated different sets of cell lines that constitutively overexpress CIC-DUX4 (Fig. 6A and Supplementary Fig. 7A, B). We used multiple cell lines, of both mouse and human origin, to exclude cell-type-specific effects. In each cell line, overexpression of CIC-DUX4 resulted in induction of ETV1, 4, and 5, which was reversible with iP300w treatment (Fig. 6B and Supplementary Fig. 7A, B) indicating the relevant functionality of CIC-DUX4 expressed in each line, and further demonstrating the effectiveness of iP300w to interfere with the CIC-DUX4/ETV4 transformation axis. We then investigated the global acetylation status of H3K18 and H3K27 after CIC-DUX4 induction. It is well accepted that acetylation of both H3 lysines is facilitated by P300/CBP and that these modifications mark active enhancers [35–37], however whether and how an increase in the global balance of acetylated to nonacetylated H3 would lead to elevated global transcription, and whether this is relevant to transformation is unclear. As we previously observed with DUX4, expression of CIC-DUX4 resulted in dramatically increased global acetylation on both lysines in all of the analyzed cell lines (Fig. 6C and Supplementary Fig. 7C). This acetylation was dependent on P300/CBP, as it was abolished in the presence of iP300w. Importantly, we also demonstrated that iP300w reduces global H3K18 and H3K27 acetylation in NCC-CDS-X1 and Kitra-SRS CDS cell lines (Fig. 6D). Taken together, this data shows that iP300w prevents and reverses CIC-DUX4-induced H3K18 and H3K27 acetylation.

## Discussion

In this study, we show that CIC-DUX4, which bears the P300-interacting DUX4 C-terminus [15], activates transcription in a way that is mechanistically related to DUX4, namely, being critically dependent on P300/CBP histone acetyltransferase activity [25]. P300 and CBP are histone acetyltransferases [38, 39] that regulate gene expression in a wide range of cellular processes, including proliferation, differentiation, response to DNA damage, senescence, and apoptosis [33, 40]. Furthermore, they interact with multiple oncogenes/tumor suppressors and are essential for their activity and carcinogenesis [41–44]. Here, we show that the oncogenic mechanism of CIC-DUX4, which involves both deregulation of cell cycle through upregulation of genes such as *CCNE1* as well as induction of invasion and metastasis potential through upregulation of *ETV4* and related Ets-family genes [16], is critically dependent on P300/CBP. Knockdown of both histone acetyltransferases P300 and CBP in CDS cell lines results in the prevention of CIC-DUX4 target gene expression leading to cell cycle arrest. Thus, we identify disruption of the CIC-DUX4/P300/CBP axis as a potential druggable approach for CDS treatment.

Currently, there are four different established CDS cell lines, NCC-CDS-X1 and NCC-CDS-X3 which originate from the same patient, NCC-CDS2-C1, and Kitra-SRS [27, 28, 45]. All have been used in multiple screens with known FDA-approved anticancer molecules. Only a handful of drugs, that are not DNA intercalators, like ponatinib, crizotinib, and bortezomib, have shown some degree of effectiveness in vitro [27, 28, 45]. By investigating downstream transcriptional targets of CIC-DUX4, Okimoto et al. identified the CCNE-CDK2 complex as a potential CDS druggable target [16]. Using dinacibil, an approved CDK2 inhibitor, they were able to suppress tumor growth and metastasis in a mouse xenograft model [16]. Applying a similar molecular approach, Nakai et al. found that the viability of Kitra-SRS cells depends on the IGF1/IGF1R pathway, which if it is interrupted with linsitinib, an IGF-1R inhibitor, results in reduced cell proliferation [27]. Both approaches deserve consideration in developing specific therapies for CDS even though they are focused on distant indirect CIC-DUX4 targets. The optimal therapy for CDS would be inactivation of CIC-DUX4, either directly or through targeting of its essential coactivators. Thus specific inhibition of P300/CBP represents a rationally-targeted pharmacological approach to the treatment of CDS.

To this end, we synthesized iP300w, a potent and selective p300/CBP inhibitor, together with several related isomers. iP300w belongs to the same spirocyclic chemical series as A-485, previously shown to be a highly selective catalytic inhibitor of P300/CBP. iP300w is structurally related to A-485, which was previously shown to be a highly selective catalytic inhibitor of P300/CBP [26]. The core of iP300w contains a spirohydantoin with (S) stereochemistry, replaced by a spirooxazolidinedione with (R) stereochemistry in A-485 (Fig. 2A, B). Initial data on earlier compounds in the spirohydantoin series showed that both (S) and (R) forms of the spirocyclic core were tolerated but a ~10-fold improvement in activity was associated with the (S) form [26]. Interestingly and surprisingly, replacing the spirohydantoin with a spirooxazolidinedione alters the stereochemical preference at this center, with a ~5-fold improvement in activity associated with (R) stereochemistry (IC50 = 0.121 μM), as opposed to (S) (IC50 = 0.025 μM) [30]. The preference for a spirooxazolidinedione rather a spirohydantoin in A-485 was attributed to a desire to reduce the number of H-bond donors, to improve permeability and ultimately oral exposure. Recent work on chemical analogs of this series aimed to further improve cellular activity of P300/CBP HAT inhibitors. In this example, researchers opted for lead compounds based on the (S)-spirohydantoin core and achieved higher oral exposure by replacing the urea moiety (present in A-485) with a methylpyrazole [32]. In agreement with Yang et al. [32], we find that the significant improvements in cellular activity can be achieved in A-485-like compounds containing a (*S*)-spirohydantoin core. Further studies to assess oral exposure and PK/PD of iP300w are warranted to fully assess the potential for clinical translation of this inhibitor.

A-485 was shown to suppress proliferation of a wide spectrum of cancer cell lines, more prominently in those of hematopoietic origin [26]. A-485 has also been found to be effective against high MITF-expressing melanoma cell lines [46] and nuclear protein of the testis (NUT) midline carcinoma [47]. Additionally, A-485 increases the sensitivity of non-small-cell lung carcinoma cells to TRAIL, or A-485 in combination with PD-L1 blockade treatment dramatically reduced prostate cancer tumor growth [48, 49]. The potential to target cancer-associated epigenetic abnormalities is a promising strategy for cancer treatment [50]. Several HDAC inhibitors, including vorinostat, belinostat, and panobinostat are FDA approved for cancer treatment and a dozen more drugs are in the late-stage clinical trials (for review see ref. [51]). However, due to the lack of a potent, selective, and metabolically stable molecule, currently, there is not yet an approved therapeutic HAT inhibitor. While the general efficacy of A-485 against the large number of tumor cell lines is impressive, none of the lines tested involved CIC-DUX4 fusions, where our data shows that P300 inhibition would specifically inactivate the primary oncogenic driver, leading to a specific toxicity in excess of the generic toxicity of inhibiting HAT activity. Furthermore, doses used in these studies are relatively high. Notably, in studies screening FDA approved drugs on CDS cells, screening was performed at 10 µM [27], while in the studies above we show that iP300w is effective at 0.003 µM. In testing against pancreatic and colorectal cancer cell lines expected to have a general P300/CBP dependence, we did not observe toxicities until concentrations of 0.3 µM and above, highlighting the extreme sensitivity of CIC-DUX4-induced cancers to iP300w. Furthermore, in our cell-based assays, iP300w was about two orders of magnitude more active than A-485. Considering that these compounds begin to show toxic effects on cells at micromolar concentrations, retaining activity down into the low sub-micromolar range will be essential, making A-485 undesirable in this regard. The greater potency of iP300w is thus critical. Indeed, the lack of toxicity at the effective concentration is highlighted by the fact that our first use of iP300w was against full length DUX4, a toxic protein that causes cell death, and this cell death was prevented by iP300w [25].

As current therapies for CDS are ineffective or very short-lived, with tumors rapidly developing resistance, the ability to inhibit the molecular driver of the disease could provide a transformative therapy for these patients. Based on the in vitro and in vivo data presented here, iP300w is therefore a very promising candidate for targeted therapy in CDS. iP300w will also likely be effective on those cancers for which A-485 has been shown active, and probably at much lower doses. Furthermore, the extreme sensitivity of CDS cell lines to iP300w vis-à-vis other cancer lines highlights the value of specifically targeting the oncogenic driver. It is likely that certain other oncogenic fusion transcription factors carry a similar p300/CBP-dependence, and where this occurs, iP300w might be similarly effective. Thus, the continued translational development of iP300w for cancer and CDS in particular is strongly warranted.

## Methods

### Synthesis of iP300w

See Supplementary Information for synthetic methods. iP300w is now commercially available from Tocris Bioscience.

### Cell culture

All of the basal media were purchased from HyClone, fetal bovine serum (FBS) was from PeakSerum (Ps-FB3, lot 293Q16), and Glutamax (Glu) and Penicillin/Streptomycin (P/S) were from GIBCO. NCC-CDS-X1 (CIC-DUX4 sarcoma cell line, a generous gift from Tadashi Kondo) cells were cultured in RPMI with 10% FBS, Glu and P/S, Kitra-SRS (CIC-DUX4 sarcoma cell line, a generous gift from Hidetatsu Otani), 3T3, 293T, and C2C12 cells were cultured in DMEM/10%FBS/Glu/P/S. Their identity as CDS cells was confirmed by PCR for CIC-DUX4. The immortalized human myoblast LHCN-M2 cell line was cultured in proliferation medium: F10 supplemented with 20% FBS, 2-mercaptoethanol 1× (GIBCO), 10^−9^ M dexamethasone (Sigma), 10 ng/mL bFGF (Peprotech), and Glu/P/S. All cells were cultured at 37 °C in a 5% CO_2_ atmosphere.

### Antibodies, western blot, and immunofluorescence

For western blot analyses, cells were lysed with RIPA buffer supplemented with protease inhibitor cocktail (Complete, Roche), and proteins were separated on 10% SDS-PAGE gels, then transferred to PVDF membranes. Antibodies were diluted in 5% skim milk in TBST and incubated overnight at 4 °C or 1 h at RT. An appropriate secondary HRP conjugated antibody was incubated for 1 h at RT. Membranes were then washed with TBST, and signal was visualized using Pierce ECL western blotting substrate (Thermo Scientific).

For immunofluorescence, cells cultured in 96-well plates were fixed in 4% PFA for 10 min, washed twice with PBS, permeabilized with 0.3% Triton X for 30 min, and blocked with 3% BSA for 1 h at R/T. Primary antibodies were diluted in 3% BSA and incubated o/n at 4 °C. An appropriate conjugated secondary antibody was applied for 60 min at RT. Nuclei were visualized using DAPI (1:5000, Sigma). Antibodies used in the study: GAPDH-HRP (1:5000, 60004, Proteintech), rabbit anti-Histone H3K18Ac (1:500, ab1191, Abcam), rabbit anti-Histone H3K27Ac (1:500, ab1791, Abcam, lot: GR3297878-1), rabbit anti-ETV4 (1:250, D2720, Santa Cruz), rabbit anti-Ki-67 (dilution 1:250, 9129T, Cell Signaling, lot: 3), rabbit anti-DUX4 (1:1000, ab124699, Abcam), secondary Alexa fluor 555 Goat Anti-Rabbit (1:500, Invitrogen), anti-rabbit CBP (1:1000, 7389S, Cell Signaling, lot: 5), anti-mouse P300 (1:500, 61401, Active Motif, lot# 31420004), HRP conjugated anti-rabbit (1:5000, 111-035-003, Jackson Immuno Research, lot: 149393), and HRP conjugated anti-mouse: (1:2500, NBP1-75130, Novus, lot 58-173-090418).

### Cell viability (ATP) assay

Cell lines were plated in a 96-well dish (1 × 10^5^ cells/well), and the following day were treated with iP300w or its stereoisomers. ATP assays were performed using CellTiter-Glo® Luminescent Cell Viability Assay (Promega) according to the manufacturer’s instructions. Luminescence was analyzed on POLARstar Optima Microplate Reader (BMG Labtech, Offenburg, Germany).

### Spheroid assay

For spheroid formation assays, 2.5 × 10^4^ NCC-CDS-X1 cells were seeded into 96-well plates (96-well Clear Flat Bottom Ultra-Low Attachment Microplate; Corning, Inc., Corning, NY, USA) in MEM/10%FBS media. Treatment with iP300w was started two days after the plating when the cells formed compact spheres. The size of the spheroids was calculated on 10× images using ImageJ.

### EdU incorporation

EdU labeling and visualization was done using Click-iT® EdU Flow Cytometry Assay Kit (Thermo Fisher Scientific). Briefly, 1 × 10^5^ NCC-CDS-X1 cells were plated in each well of a 24-well plate. The following day, continual treatment with 0.3 µM iP300w was initiated. For the pulse experiment, cells were treated with iP300w for 4 or 24 h and analyzed 24 h later. Cells were cultured with EdU (10 nM) in the last 4 h of the experiment, and samples were prepared for analyses according to the manufacture’s instruction. FACS analyses were performed on a BD FACSAria instrument and data was analyzed using FlowJo (BD Biosciences). Experiments were performed on at least three biological replicates.

### Mouse tumor formation and evaluation of iP300w in vivo

Mice were maintained, and in vivo experiments were conducted at the University of Minnesota Research Animal Resources facility, under a protocol (1903-36866A) approved by IACUC. Mice were divided into two groups, matching sex and age as closely as possible, and the control vs. experimental group decided by lot. Three-months-old immunodeficient NSG-MDX mice (*n* = 8) were transplanted with 1 × 10^7^ NCC-CDS-X1 cells resuspended in 100 µL of a mixture of medium and Matrigel (Corning). Visible tumor masses at the sites of injection were detected on day 9 after transplantation. All of the injected mice developed tumors. At this point, the mice were randomized into two groups, control and iP300w treated, and initial measurement of the tumors was performed. iP300w was initially dissolved in DMSO at 10 mM and then diluted in 100 µL PBS. Each mouse received 1.4 mg/kg iP300w intraperitoneally twice daily or vehicle in the control group. The dose of iP300w was determined based on the effective iP300w concentration on CDS sarcomas in vitro (1 µM) and from our previous experience with iP300w and DUX4 inactivation in the iDUX4pA mouse, an FSHD animal model [25]. Tumor size was recorded every 4 days and the volume was calculated using the formula (length × width^2^)/2. Mice were identified by number. Investigator was blind to the status of the particular mouse during the dissection, weighing, and photographing of the tumors.

### Generation of CIC-DUX4 expression cell lines

Viral supernatants were produced in 293T cells. For transducing mouse cells (3T3 and C2C12), the CIC-DUX4 expression vector [17] was packaged with pCL-Eco, and pVSV-G/pCMVgp were used for infecting human cells (LHCN-M2). Packaging cells were transfected with DNA plasmids using TransIT-LT1 reagent (Mirus). Viral supernatant was collected at 48 and 72 h of post-transfection and applied to the cells. Two days of post-infection GFP positive cells were sorted using a BD FACSAria II. Established cell lines were tested for CIC-DUX4 expression by immunofluorescence and RT-qPCR.

### RNA isolation, quantitative real-time RT-PCR (RT-qPCR), and RNAseq

RNA was extracted using an RNA extraction kit (Zymo) and cDNA was made using 0.5 µg total RNA with oligo-dT primer and Verso cDNA Synthesis Kit (Thermo Scientific) following the manufacturer’s instructions. qPCRs were performed by using Premix Ex Taq (Probe qPCR, Takara) or SYBR-green. Probes and primer sets used in this study are presented in Supplementary Table 2. Gene expression levels were normalized to that of *GAPDH or B2M* and analyzed with 7500 System Software using the ∆CT method (Applied Biosystems).

RNA-seq library preparation was done with 500 ng total RNA from NCC-CDS-X1 cells treated for 4 or 24 h with iP300w (0.3 µM) using the Swift Rapid RNA Library Kit (SwiftBioscience). Thirty-six base paired-end sequencing was performed on an Illumina NextSeq instrument at the University of Minnesota Genomics Center.

### RNA interference

NCC-CDS-X1 and Kitra-SRS cells were seeded into 96-well plates (5 × 10^4^/well) for cell viability evaluation or a 24-well plate (1.5 × 10^5^/well) for RNA isolation. The following day, 50 nM siRNA for human P300 (L-003486-00-0005), CREBBP (L-003477-00-0005), or scrambled control (SMARTpool, Dharmacon) were transfected using Lipofectamine RNAiMAX (Invitrogen). RNA was isolated 48 h of post-transfection and the effect on cell viability was analyzed at 72 and 96 h of post-transfection.

### Bioinformatics

Paired-end Illumina sequencing reads were trimmed with TrimGalore (0.6.0) and transcript abundance was quantified using the human Gencode annotations (v34) using Salmon (v 1.2.1) with the GC-bias correction option. Counts were imported into R (v 4.0.2) using tximeta (v 1.6.3). Differentially expressed genes were identified with DESeq2 (v 1.28.1) and figures were made with the ComplexHeatmap (v 2.4.3), cluster Profiler (v 3.16.1) and ggplot2 (v 3.3.2) R packages. Sequencing reads and processed data have been deposited into GEO under the accession number GSE165729.

### Statistics

Graphpad Prism software was used for statistical analyses of the data, except when indicated. The sample size was chosen according to prior experience with the assays used to ensure adequate statistical power. Variance was similar within groups. Differences between groups were evaluated by one-way or two-way analysis of variance (ANOVA) followed by Tukey’s post-hoc tests. Differences were considered significant at *p*-values of 0.05 or lower.

## Supplementary information

Supplementary Figure 1

Supplementary Figure 2

Supplementary Figure 3

Supplementary Figure 4

Supplementary Figure 5

Supplementary Figure 6

Supplementary Figure 7

Supplementary Table 1

Supplementary Table 2

Crystal structure iP300v

Crystal structure Comp 6

Supplementary Methods

Supplementary western blots

CTs of qPCRs
