# Supplementary material for: Inactivation of the CIC-DUX4 oncogene through P300/CBP inhibition, a therapeutic approach for CIC-DUX4 sarcoma
Source: Oncogenesis. 2021 Oct 12;10(10):68. doi: 10.1038/s41389-021-00357-4 (PMC8511258; doi:10.1038/s41389-021-00357-4)
Supplement: Supplementary file 1 — Supplementary Figure 1 [file 41389_2021_357_MOESM1_ESM.pptx]

## Slide 1
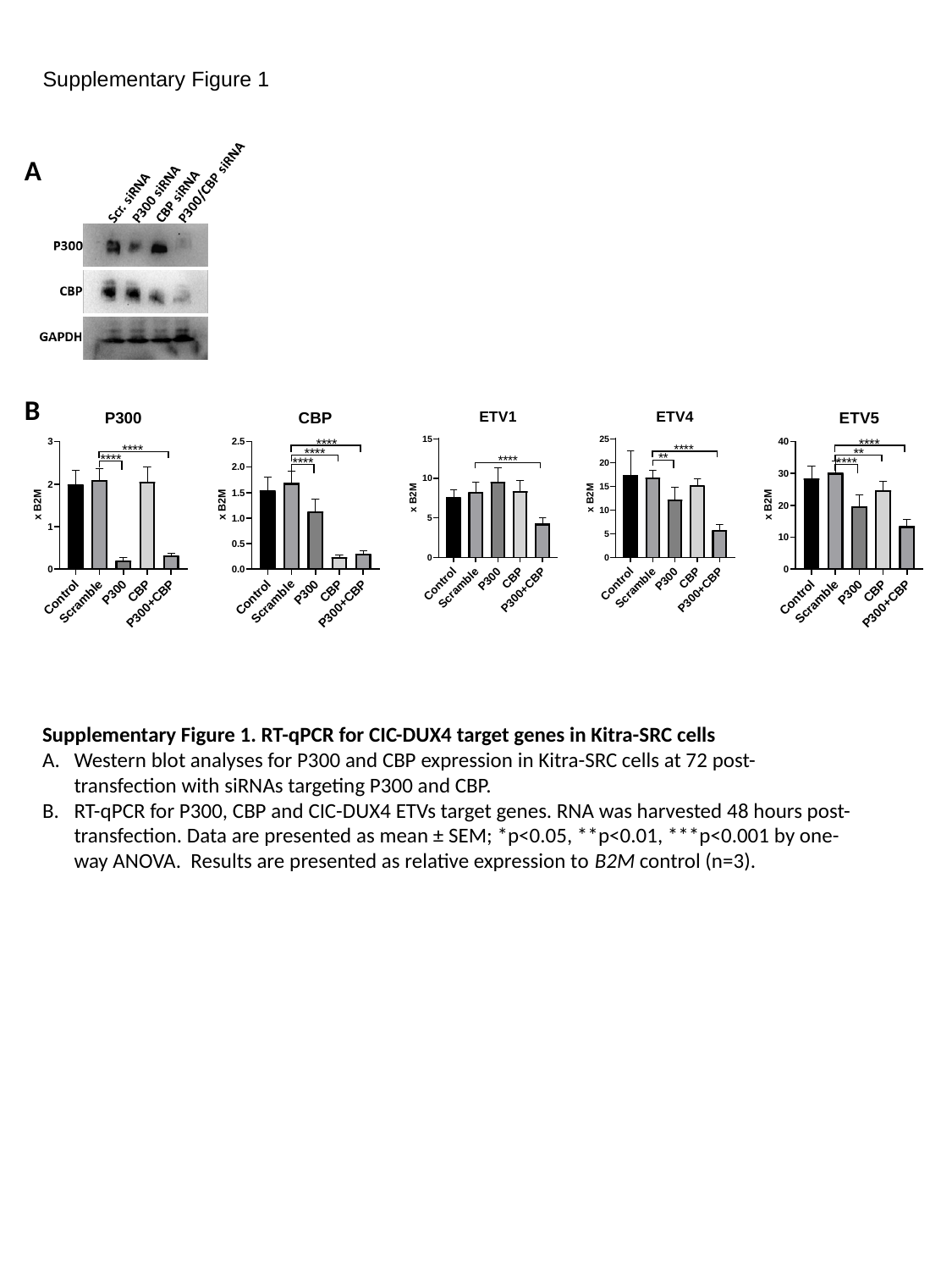

Supplementary Figure 1
A
B
Supplementary Figure 1. RT-qPCR for CIC-DUX4 target genes in Kitra-SRC cells
Western blot analyses for P300 and CBP expression in Kitra-SRC cells at 72 post- transfection with siRNAs targeting P300 and CBP.
RT-qPCR for P300, CBP and CIC-DUX4 ETVs target genes. RNA was harvested 48 hours post-transfection. Data are presented as mean ± SEM; *p<0.05, **p<0.01, ***p<0.001 by one-way ANOVA. Results are presented as relative expression to B2M control (n=3).
