# Supplementary material for: Inactivation of the CIC-DUX4 oncogene through P300/CBP inhibition, a therapeutic approach for CIC-DUX4 sarcoma
Source: Oncogenesis. 2021 Oct 12;10(10):68. doi: 10.1038/s41389-021-00357-4 (PMC8511258; doi:10.1038/s41389-021-00357-4)
Supplement: Supplementary file 2 — Supplementary Figure 2 [file 41389_2021_357_MOESM2_ESM.pdf]

Supplementary Figure 2

A

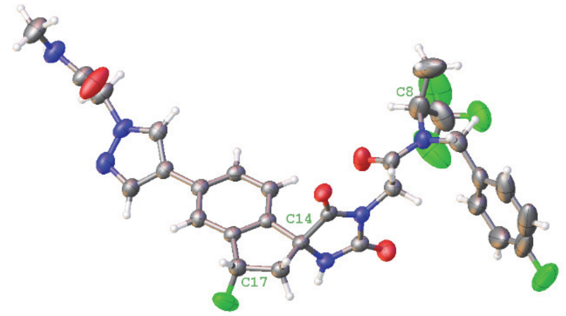

B

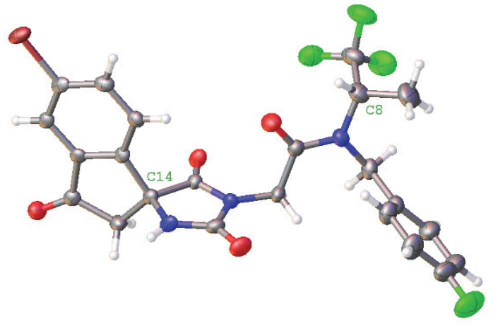

Supplementary Figure 2. Crystal structures of compounds (**14**) and (**6**).

A. Crystal Structure of iP300v (**14**). Crystals of iP300v were grown from a MeOH solution via slow evaporation. X-Ray diffraction analysis showed that crystals of iP300v contain MeOH, disordered over 2 positions. Absolute configuration has been determined by anomalous dispersion (Flack parameter = 0.02(5)) to be C8 (S), C14 (R) and C17 (S).

B. Crystal Structure of compound (**6**). Crystals of (**6**) were grown from a DMSO solution via slow evaporation. X-Ray diffraction analysis showed that (**6**) crystallises with Z' = 2. Absolute configuration has been determined by anomalous dispersion (Flack parameter = -0.052(13)) to be C8 (S), C14 (S).
