# Supplementary material for: Inactivation of the CIC-DUX4 oncogene through P300/CBP inhibition, a therapeutic approach for CIC-DUX4 sarcoma
Source: Oncogenesis. 2021 Oct 12;10(10):68. doi: 10.1038/s41389-021-00357-4 (PMC8511258; doi:10.1038/s41389-021-00357-4)
Supplement: Supplementary file 3 — Supplementary Figure 3 [file 41389_2021_357_MOESM3_ESM.pptx]

## Slide 1
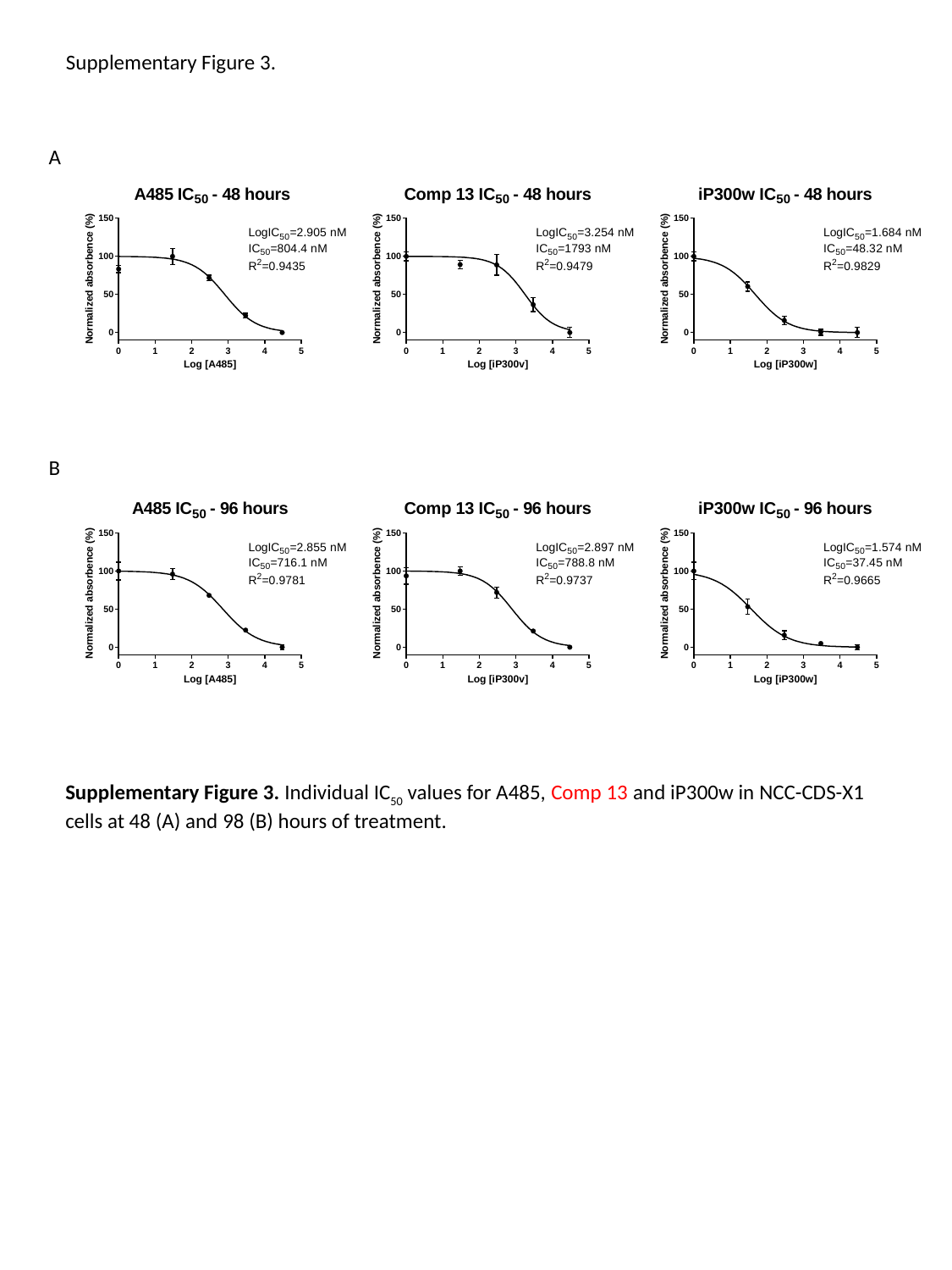

Supplementary Figure 3.
A
B
Supplementary Figure 3. Individual IC50 values for A485, Comp 13 and iP300w in NCC-CDS-X1 cells at 48 (A) and 98 (B) hours of treatment.
