# Supplementary material for: Inactivation of the CIC-DUX4 oncogene through P300/CBP inhibition, a therapeutic approach for CIC-DUX4 sarcoma
Source: Oncogenesis. 2021 Oct 12;10(10):68. doi: 10.1038/s41389-021-00357-4 (PMC8511258; doi:10.1038/s41389-021-00357-4)
Supplement: Supplementary file 4 — Supplementary Figure 4 [file 41389_2021_357_MOESM4_ESM.pdf]

Supplementary Figure 4

A

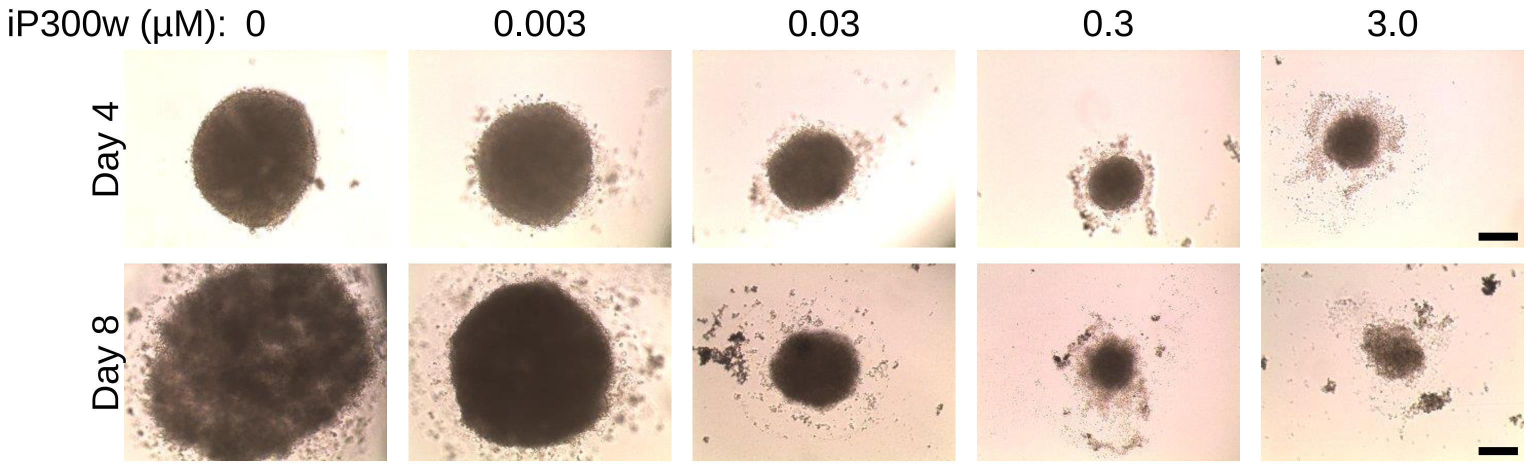

B

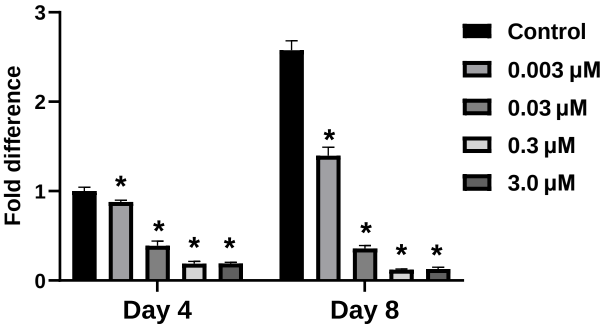

Supplementary Figure 4. Morphology of NCC-CDS-X1 spheroids at day 4 and 8 of treatment.

A. Representative picture of spheroids treated with various concentration of iP300w. Scale bar 50 µm.

B. Summary of the size of the spheroids presented in “A”. Data were presented as fold difference to the control at day 4, n=6. Statistical significance was calculated by two-way ANOVA.
