# Supplementary material for: Inactivation of the CIC-DUX4 oncogene through P300/CBP inhibition, a therapeutic approach for CIC-DUX4 sarcoma
Source: Oncogenesis. 2021 Oct 12;10(10):68. doi: 10.1038/s41389-021-00357-4 (PMC8511258; doi:10.1038/s41389-021-00357-4)
Supplement: Supplementary file 5 — Supplementary Figure 5 [file 41389_2021_357_MOESM5_ESM.pptx]

## Slide 1
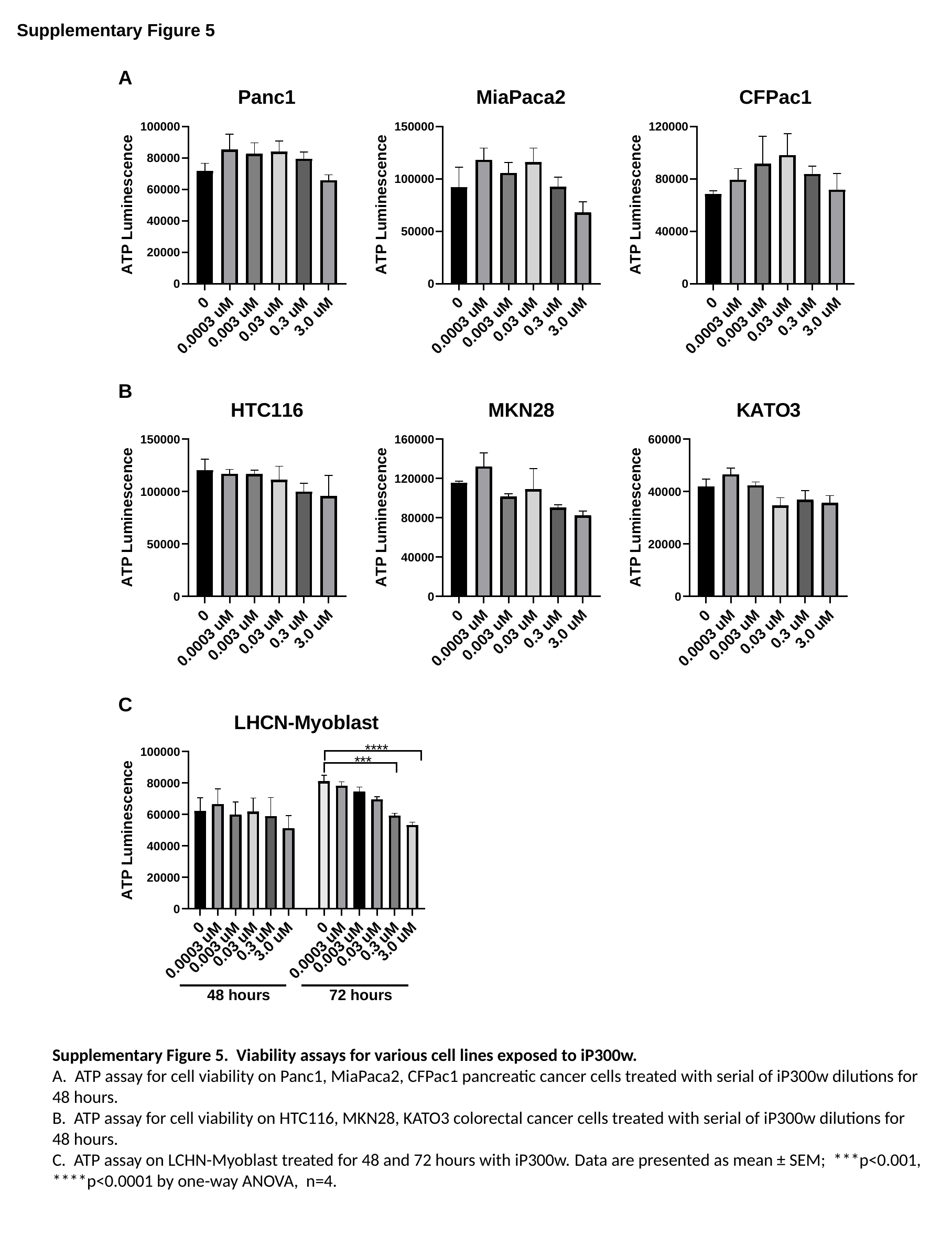

Supplementary Figure 5
A
B
C
Supplementary Figure 5. Viability assays for various cell lines exposed to iP300w.
A. ATP assay for cell viability on Panc1, MiaPaca2, CFPac1 pancreatic cancer cells treated with serial of iP300w dilutions for 48 hours.
B. ATP assay for cell viability on HTC116, MKN28, KATO3 colorectal cancer cells treated with serial of iP300w dilutions for 48 hours.
C. ATP assay on LCHN-Myoblast treated for 48 and 72 hours with iP300w. Data are presented as mean ± SEM; ***p<0.001, ****p<0.0001 by one-way ANOVA, n=4.
