# Supplementary material for: Inactivation of the CIC-DUX4 oncogene through P300/CBP inhibition, a therapeutic approach for CIC-DUX4 sarcoma
Source: Oncogenesis. 2021 Oct 12;10(10):68. doi: 10.1038/s41389-021-00357-4 (PMC8511258; doi:10.1038/s41389-021-00357-4)
Supplement: Supplementary file 6 — Supplementary Figure 6 [file 41389_2021_357_MOESM6_ESM.pdf]

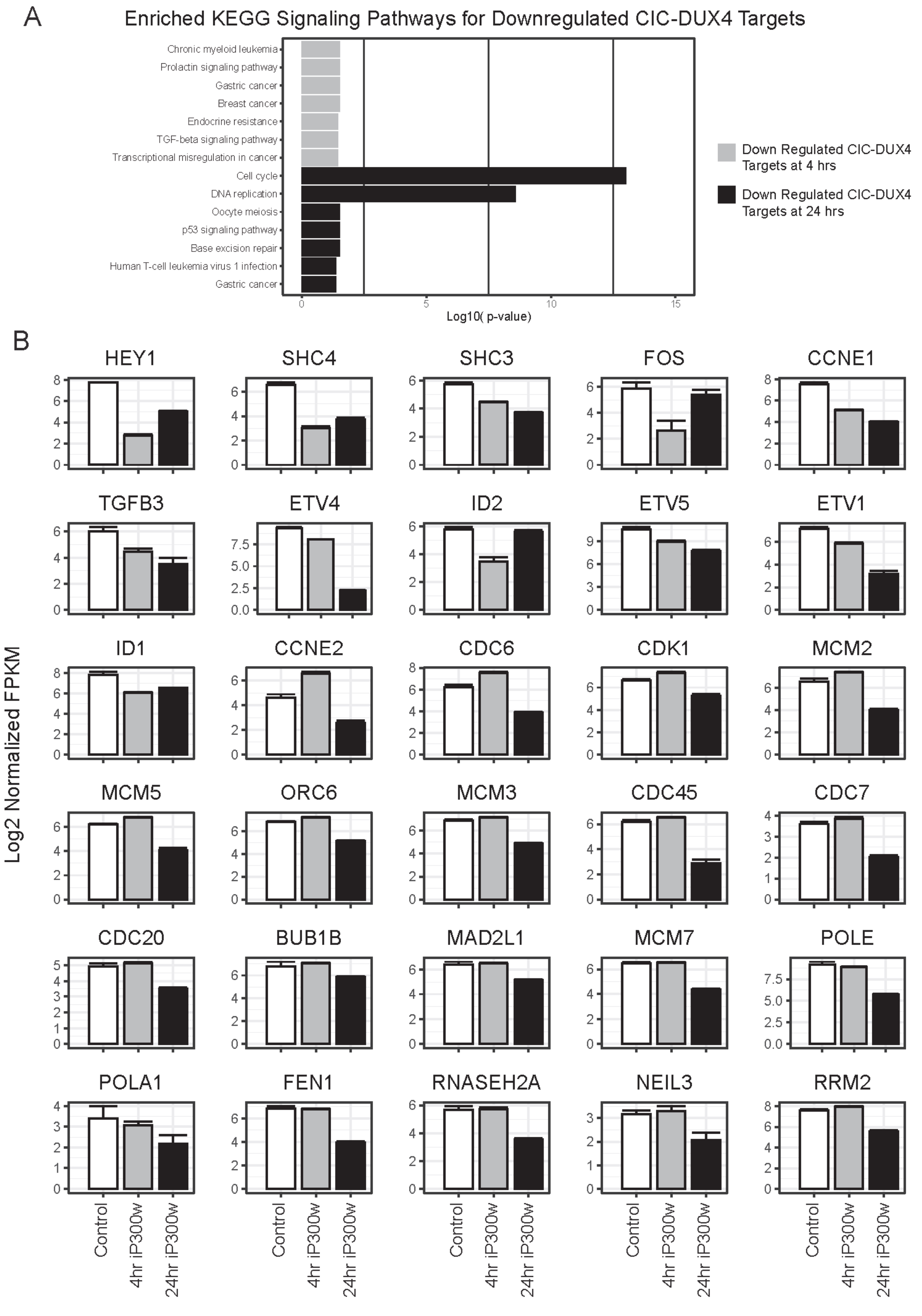

Supplementary Figure 6. Pathway Analysis for targets of CIC-DUX4 downregulated upon treatment with iP300w.

A. Enriched KEGG pathways (hypergeometric test, Benjamini-Hochberg adjusted p-value < 0.05) of the downregulated genes at 4 hours (gray) and 24 hours (black) that have previously been identified as CIC-DUX4 targets (Okimoto et al., 2019).

B. Barplots of log2 transformed FPKM values for genes in enriched KEGG pathways in (A) for the control (white), 4 hours (gray) and 24 hours (black) conditions.
