# Supplementary material for: Inactivation of the CIC-DUX4 oncogene through P300/CBP inhibition, a therapeutic approach for CIC-DUX4 sarcoma
Source: Oncogenesis. 2021 Oct 12;10(10):68. doi: 10.1038/s41389-021-00357-4 (PMC8511258; doi:10.1038/s41389-021-00357-4)
Supplement: Supplementary file 7 — Supplementary Figure 7 [file 41389_2021_357_MOESM7_ESM.pdf]

Supplementary Figure 7

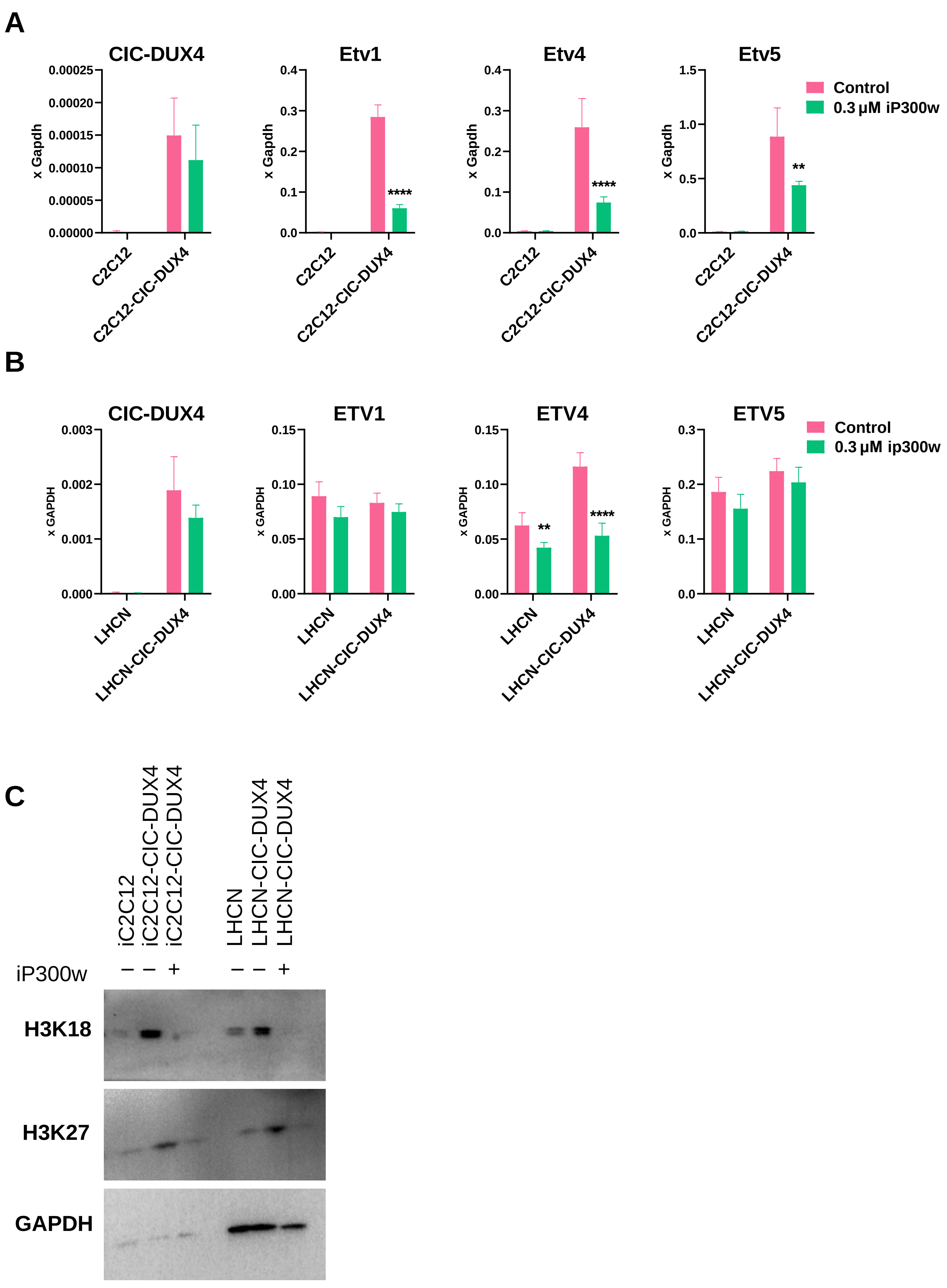

Supplementary Figure 7. Effects of iP300w on mouse and human myoblast cell lines.

A. RT-qPCR for CIC-DUX4 target genes in C2C12-CIC-DUX4 mouse myoblasts treated with 0.3 µM iP300w for 18 hours.
B. RT-qPCR for CIC-DUX4 target genes in LHCN-CIC-DUX4 human myoblasts treated with 0.3 µM iP300w for 18 hours.
C. Western blots for acetylated H3K18 and H3K27 in the conditions presented in A and B, above.
