# Supplementary material for: Inactivation of the CIC-DUX4 oncogene through P300/CBP inhibition, a therapeutic approach for CIC-DUX4 sarcoma
Source: Oncogenesis. 2021 Oct 12;10(10):68. doi: 10.1038/s41389-021-00357-4 (PMC8511258; doi:10.1038/s41389-021-00357-4)
Supplement: Supplementary file 9 — Supplementary Table 2 [file 41389_2021_357_MOESM9_ESM.docx]

Supplementary Table 2. Primer sequences used in RTqPCR assays.

| **Gene** | **Direction** | **Sequence** |
| --- | --- | --- |
| CCNE1 | F | 5’ - TTT TTG CAG GAT CCA GAT GA - 3’ |
|  | R | 5’ - TGC ACG TTG AGT TTG GGT AA - 3’ |
| CIC-DUX4 | F | 5’ - CGC TGT GTG GAG TCT CTC ACC CG - 3’ |
|  | R | 5’- GAG GAC GTG CTT GGG GAG CTA CAG - 3’ |
| CBP | F | 5’ - GTC CAG TTG CCA CAA GCA C - 3’ |
|  | R | 5’ - CAT TCG GGA AGG AGA AAT GG - 3’ |
| ETV1 | F | 5’ - TAC CCC ATG GAC CAC AGA TT – 3 |
|  | R | 5’ - CAC TGG GTC GTG GTA CTC CT – 3 |
| ETV4 | F | 5’ - GTC ACT TCC AGG AGA CGT GG – 3 |
|  | R | 5’ - ATA GGC ACT GGA GTA AAG GCA C – 3 |
| ETV5 | F | 5’ - TCT GAG CTG TCG TCT TGT AGC C – 3 |
|  | R | 5’ - GTT ATT GGC TTG AAC CCA GAG G – 3 |
| EP300 | F | 5’ - GGC TGT ATC AGA GCG TAT TGT C – 3 |
|  | R | 5’ - CCT CGA AAT AAG GCA ATT CC – 3 |
| CCNE1 | F | 5’ - TTT TTG CAG GAT CCA GAT GA - 3’ |
|  | R | 5’ - TGC ACG TTG AGT TTG GGT AA - 3’ |
| CCNE2 | F | 5’ - GGG AAA CAT TTT ATC TTG CAC A - 3’ |
|  | R | 5’ - CTG CAA GCA CCA TCA GTG AC - 3’ |
| CDK2 | F | 5’ - TAC CAC AGG GTC ACC ACC TC - 3’ |
|  | R | 5’ - TCC TCC ACC GAG ACC TTA AA - 3’ |
| GAPDH | Hs99999905_m1 | |
| B2M | Hs00187842_m1 | |

**Mouse Genes**

| **Gene** | **Direction** | **Sequence** |
| --- | --- | --- |
| Etv1 | F | 5’ - TCC TGG CTC ATC CAA GCA GAA C - 3’ |
|  | R | 5’ - CGG TAC ATT CCA GGC TCT TGC T - 3’ |
| Etv4 | F | 5’ - CCA CCA GGA TCA AGA AGG AA - 3’ |
|  | R | 5’ - CCC TGA GGA GAT GTG AAG GA - 3’ |
| Etv5 | F | 5’ - AGC CCA CCA TGT ATC GAG AG - 3’ |
|  | R | 5’ - TGC ATG ATG CCC TTT TCA TA - 3’ |
| Gapdh | Mm99999915_g1 | |
