# Supplementary material for: Inactivation of the CIC-DUX4 oncogene through P300/CBP inhibition, a therapeutic approach for CIC-DUX4 sarcoma
Source: Oncogenesis. 2021 Oct 12;10(10):68. doi: 10.1038/s41389-021-00357-4 (PMC8511258; doi:10.1038/s41389-021-00357-4)
Supplement: Supplementary file 12 — Supplementary Methods [file 41389_2021_357_MOESM12_ESM.docx]

**SUPPLEMENTARY METHODS**

**Suppression of the CIC-DUX4 oncogene through p300/CBP inhibition**

***Synthesis of iP300w and diastereoisomers***

**General Methods**

All reagents and solvents were purchased from commercial sources and used without further purification. Nuclear magnetic resonance spectra were recorded on a Bruker Avance III HD spectrometer operating at 400 MHz for ^1^H NMR, 100 MHz for ^13^C NMR and 376 MHz for ^19^F NMR. ^1^H NMR and ^13^C NMR chemical shifts (δ) are reported in parts per million (ppm) and are referenced to residual protium in solvent and to the carbon resonances of the residual solvent peak respectively. DEPT and correlation spectra were run in conjunction to aid assignment. ^19^F NMR chemical shifts are reported in ppm and are uncorrected. Coupling constants (*J*) are quoted in Hertz (Hz), and the following abbreviations were used to report multiplicity: s= singlet, d= doublet, dd= doublet of doublets, ddd= double doublet of doublets, t= triplet, q= quartet, m= multiplet, br s= broad singlet.

Purification by flash column chromatography was carried out using Fisher Scientific silica gel 60Å (35-70 μm). Analytical thin layer chromatography was performed on glass plates pre-coated with silica gel (Analtech, UNIPLATE™ 250 μm / UV254), with visualization being achieved using UV light (254 nm) and/or by staining with alkaline potassium permanganate dip.

Reaction monitoring LC-MS analyses were conducted using Agilent InfinityLab LC/MSD systems. Chiral GC analysis was conducted using an Agilent 7890A GC system. Optical rotations were recorded on a Bellingham & Stanley ADP450 polarimeter. High resolution mass spectral (HRMS) data was collected in the laboratories of the University of Bath Chemistry Department using an Agilent 6545 LC/Q-TOF system. Single crystal X-ray measurements were recorded by Indicatrix Crystallography Ltd. in the laboratories of Newcastle University Chemistry Department.

**5'-Bromo-2',3'-dihydrospiro[imidazolidine-4,1'-indene]-2,5-dione (1)**

A stirred suspension of 5-bromoindan-1-one (60.00 g, 284.29 mmol) in ethanol (246 mL) and water (164 mL) at ambient temperature was treated with ammonium carbonate (83.86 g, 872.76 mmol) and potassium cyanide (27.77 g, 426.43 mmol). The reaction mixture was heated to 70 °C and stirred under a flow of nitrogen at this temperature for a total of 161 hours. During this period it was found to be necessary three times to cool and recharge with further portions of ammonium carbonate (1 eq) and potassium cyanide (0.5 eq) before raising the temperature again. Upon completion of the reaction, and after cooling to ambient temperature, the reaction mixture was treated with water (700 mL) and ethyl acetate (500 mL). The mixture was filtered, and the layers of the filtrate separated. The aqueous component was extracted with ethyl acetate (2 × 200 mL), and the combined organics were washed with brine, dried over anhydrous magnesium sulfate and concentrated under reduced pressure. Purification by dry flash chromatography, eluting with 2-4% MeOH/DCM, afforded the title compound **(1)** as a light brown solid (42.80 g, 54%).

^1^H NMR (DMSO-d_6_) δ: 10.81 (1H, br s), 8.44 (1H, br s), 7.55 (1H, d, *J*= 1.2), 7.43 (1H, dd, *J*= 8.4, 1.2), 7.12 (1H, d, *J*= 8.4), 3.07-2.94 (2H, m), 2.56-2.50 (1H, m), 2.21-2.13 (1H, m).

^13^C NMR (DMSO-d_6_) δ: 176.7, 156.4, 146.7, 140.7, 129.9, 127.8, 124.6, 122.0, 71.2, 36.4, 29.4.

m/z (ES^-^): 279.1, 281.1 [M-H^+^]^-^

**5'-Bromospiro[imidazolidine-4,1'-indene]-2,3',5(2'H)-trione (2)**

A stirred mixture of 5'-bromo-2',3'-dihydrospiro[imidazolidine-4,1'-indene]-2,5-dione **(1)** (38.70 g, 137.67 mmol), tetrabutylammonium hydrogen sulfate (9.35 g, 27.53 mmol) and sodium 2-iodobenzenesulfonic acid (2.11 g, 6.88 mmol) in acetonitrile (1450 mL) at ambient temperature was treated with potassium peroxymonosulfate (126.95 g, 413.01 mmol) in portions. The reaction mixture was heated to 65 °C and stirred for a total of 56 hours. During this period it was found necessary to periodically cool and recharge with further portions of potassium peroxymonosulfate (eventually up to a total of 8 equivalents were added) before raising the temperature again. Upon completion of the reaction, and after cooling to ambient temperature, the reaction mixture was filtered and washed with acetone (900 mL). The collected solid was treated with hot acetone (5 × 500 mL), these extracts being concentrated to a solid and triturated with ethyl acetate to give an initial crop of product. All the filtrates were combined and concentrated and purified by flash column chromatography, eluting with 3% MeOH/DCM, to afford a second crop of product. This process afforded the title compound **(2)** as a white solid (22.15 g, 55%).

^1^H NMR (DMSO-d_6_) δ: 11.19 (1H, br s), 8.56 (1H, br s), 7.96 (1H, dd, *J*= 8.2, 2.0), 7.90 (1H, d, *J*= 2.0), 7.62 (1H, d, *J*= 8.2), 3.15 (1H, d, *J*= 18.6), 2.92 (1H, d, *J*= 18.6).

^13^C NMR (DMSO-d_6_) δ: 199.6, 175.8, 157.0, 150.6, 139.0, 138.6, 127.0, 126.0, 124.3, 65.7, 47.3.

m/z (ES^+^): 295.1, 297.1 [M+H^+^]^+^

**(*S*)-1,1,1-Trifluoro-*N*-(4-fluorobenzyl)propan-2-amine (3)**

To a stirred solution of (2*S*)-1,1,1-trifluoropropan-2-amine hydrochloride (34.50 g, 230.70 mmol, [α]_D_^22^ = +5.8° (c=1.0, MeOH)) and potassium carbonate (127.54 g, 922.81 mmol) in DMF (345 mL) at ambient temperature was added 1-(bromomethyl)-4-fluoro-benzene (34.50 mL, 276.84 mmol) in a dropwise fashion over a period of 10 minutes, and the resulting reaction mixture was stirred for 18 hours. The reaction mixture was poured into water (2.8 L) and extracted with ethyl acetate (3 × 1 L). The combined organic extracts were washed with water (3 × 1 L), dried over anhydrous magnesium sulfate, and concentrated under reduced pressure to give a colourless oil. Purification by flash column chromatography, eluting with 4% ethyl actetate/petroleum ether (40:60), afforded the title compound **(3)** as a colourless oil (30.79 g, 60%).

^1^H NMR (CDCl_3_) δ: 7.33-7.28 (2H, m), 7.04-6.99 (2H, m), 3.93-3.84 (2H, m), 3.22-3.11 (1H, m), 1.26-1.24 (3H, m).

^13^C NMR (CDCl_3_) δ: 162.1 (d, *J*_CF_= 243.6), 135.3 (d, *J*_CF_= 3.0), 129.6 (d, *J*_CF_= 7.9), 127.0 (q, *J*_CF_= 281.3), 115.3 (d, *J*_CF_= 21.1), 54.0 (q, *J*_CF_= 28.2), 50.6, 14.65 (q, *J*_CF_= 2.4).

^19^F NMR (CDCl_3_) δ: -76.45, -115.58.

Chiral GC (β-DEX™ 120, 30 m x 250 mm, 0.25 μm): 99.7%*ee.*

[α]_D_^26^ = +22.5° (c = 1, CHCl_3_).

**(*S*)-2-Bromo-*N*-(4-fluorobenzyl)-*N*-(1,1,1-trifluoropropan-2-yl)acetamide (4)**

To a stirred solution of (*S*)-1,1,1-trifluoro-*N*-(4-fluorobenzyl)propan-2-amine **(3)** (30.50 g, 137.89 mmol) in DCM (350 mL) at ambient temperature was added a solution of bromoacetyl bromide (55.66 g, 275.78 mmol) in DCM (50 mL) in a dropwise fashion over a period of 15 minutes, and the resulting reaction mixture was stirred at ambient temperature for 2 hours. After treatment with NaHCO_3_ (sat. aq.) (470 mL) and a further hour of stirring, the organic phase was separated. The aqueous component was extracted with DCM (2 × 200mL), and the combined organics were washed with NaHCO_3_ (sat. aq.) (250 mL), brine (250 mL), dried over anhydrous magnesium sulfate and concentrated under reduced pressure to give a pale yellow oil. Purification by flash column chromatography, eluting with 4-10% ethyl actetate/petroleum ether (40:60), afforded the title compound **(4)** as a colourless oil (16.90 g, 36%).

^1^H NMR (DMSO-d_6_) δ: 7.32-7.10 (4H, m), 5.34-5.28 (0.5H, m), 4.96-4.88 (0.5H, m), 4.81-4.66 (1.5H, m), 4.56 (0.5H, d, *J*= 12.0), 4.38 (0.5H, d, *J*= 16.4), 4.26 (0.5H, d, *J*= 12.0), 4.09-3.95 (1H, m), 1.39-1.29 (3H, m).

*Peaks in ^1^H NMR are split due to the presence of rotamers.*

**2-(5'-Bromo-2,3',5-trioxo-2',3'-dihydrospiro[imidazolidine-4,1'-inden]-1-yl)-N-(4-fluorobenzyl)-N-((*S*)-1,1,1-trifluoropropan-2-yl)acetamide (5)**

To a stirred solution of 5'-bromospiro[imidazolidine-4,1'-indene]-2,3',5(2'H)-trione **(2)** (22.70 g, 76.93 mmol) and potassium carbonate (21.26 g, 153.85 mmol) in DMF (200 mL) at 2 °C was dropwise added a solution of (*S*)-2-bromo-*N*-(4-fluorobenzyl)-*N*-(1,1,1-trifluoropropan-2-yl)acetamide **(4)** (26.32 g, 76.93 mmol) in DMF (50 mL) at such a rate so as to keep the temperature below 5 °C during the course of the addition. The reaction mixture was then allowed to warm to ambient temperature and stirred for 6 hours before being partitioned between water (450 mL) and ethyl acetate (450 mL). The organic phase was separated, and the aqueous component was extracted with ethyl acetate (3 × 200 mL). The combined organics were washed with brine (4 × 200 mL), dried over anhydrous magnesium sulfate and concentrated under reduced pressure. Purification by flash column chromatography, eluting with 1-2% MeOH/DCM afforded an off-white solid which was triturated with diethyl ether/petroleum ether (40:60) (1:1) to give the title compound **(5)** as a white solid (33.08 g, 77%). Separation by preparative chromatography afforded the two diastereomers **(6)** and **(7)**.

**2-((*S*)-5'-Bromo-2,3',5-trioxo-2',3'-dihydrospiro[imidazolidine-4,1'-inden]-1-yl)-*N*-(4-fluorobenzyl)-*N*-((*S*)-1,1,1-trifluoropropan-2-yl)acetamide (6)**

^1^H NMR (DMSO-d_6_, 120 °C) δ: 8.58 (1H, br s), 7.98-7.92 (1H, m), 7.88-7.86 (1H, m), 7.67-7.63 (1H, m), 7.36-7.33 (2H, m), 7.13 (2H, t, *J*= 8.8), 5.22-5.15 (1H, m), 4.82 (1H, d, *J*= 17.6), 4.65-4.50 (2H, m), 4.35-4.28 (1H, m), 3.13 (1H, d, *J*= 18.6), 2.98 (1H, d, *J*= 18.6), 1.37 (3H, d, *J*= 7.2).

m/z (ES^+^): 578.1, 580.1 [M+Na^+^]^+^

**2-((*R*)-5'-Bromo-2,3',5-trioxo-2',3'-dihydrospiro[imidazolidine-4,1'-inden]-1-yl)-*N*-(4-fluorobenzyl)-*N*-((*S*)-1,1,1-trifluoropropan-2-yl)acetamide (7)**

^1^H NMR (DMSO-d_6_, 120 °C) δ: 8.58 (1H, br s), 7.97 (1H, dd, *J*= 8.3, 1.6), 7.87 (1H, dd, *J*= 1.6, 0.4), 7.66 (1H, dd, *J*= 8.3, 0.4), 7.37-7.33 (2H, m), 7.16-7.11 (2H, m), 5.23-5.15 (1H, m), 4.83 (1H, d, *J*= 17.6), 4.64-4.50 (2H, m), 4.35-4.28 (1H, m), 3.13 (1H, d, *J*= 18.6), 2.98 (1H, d, *J*= 18.6), 1.37 (3H, d, *J*= 7.2).

m/z (ES^+^): 578.1, 580.1 [M+Na^+^]^+^

***N*-(4-Fluorobenzyl)-2-((*S*)-5'-(1-(2-(methylamino)-2-oxoethyl)-1*H*-pyrazol-4-yl)-2,3',5-trioxo-2',3'-dihydrospiro[imidazolidine-4,1'-inden]-1-yl)-*N*-((*S*)-1,1,1-trifluoropropan-2-yl)acetamide (8)**

To a stirred solution of 2-((*S*)-5'-bromo-2,3',5-trioxo-2',3'-dihydrospiro[imidazolidine-4,1'-inden]-1-yl)-*N*-(4-fluorobenzyl)-*N*-((*S*)-1,1,1-trifluoropropan-2-yl)acetamide **(6)** (3.00 g, 5.39 mmol) and *N*-methyl-2-(4-(4,4,5,5-tetramethyl-1,3,2-dioxaborolan-2-yl)-1H-pyrazol-1-yl)acetamide (1.79 g, 6.74 mmol) in 1,4-dioxane (45 mL) was added water (9 mL). The solution was purged with nitrogen, and potassium carbonate (2.24 g, 16.18 mmol) and 1,1'-bis(diphenylphosphino)ferrocenedichloropalladium(II) DCM complex (0.35 g, 0.43 mmol) were added. The reaction mixture was transferred to a sealed stainless-steel vessel and stirred and heated at 95 °C for 12 hours before being cooled to ambient temperature and concentrated under reduced pressure. The residue was treated with DCM/MeOH (3:1) and filtered, the filter cake being washed further with DCM/MeOH (3:1), and the filtrate was concentrated under reduced pressure. Purification by flash column chromatography, eluting with 3-4% (MeOH + 5% NH_4_OH)/DCM, afforded a beige solid. Trituration with diethyl ether afforded the title compound **(8)** as an off-white solid (2.56 g, 77%).

^1^H NMR (DMSO-d_6_, 120 °C) δ: 8.57 (1H, s), 8.23 (1H, d, *J*= 0.6), 7.99 (1H, dd, *J* = 8.0, 1.7), 7.96 (1H, d, *J* = 0.7), 7.87 (1H, m), 7.67 (1H, dd, *J* = 8.1, 0.7), 7.54 (1H, br s), 7.40 – 7.33 (2H, m), 7.14 (2H, t, *J* = 8.7), 5.28 – 5.14 (1H, m), 4.84 (1H, d, *J* = 17.7), 4.80 (2H, s), 4.64 (1H, d, *J*= 17.7), 4.52 (1H, d, *J*= 17.0), 4.32 (1H, d, *J* = 17.0), 3.04 (2H, dd, *J* = 37.0, 17.9), 2.69 (3H, d, *J* = 4.7), 1.38 (3H, d, *J* = 7.1).

m/z (ES^+^): 615.3 [M+H^+^]^+^

***N*-(4-Fluorobenzyl)-2-((*R*)-5'-(1-(2-(methylamino)-2-oxoethyl)-1*H*-pyrazol-4-yl)-2,3',5-trioxo-2',3'-dihydrospiro[imidazolidine-4,1'-inden]-1-yl)-*N*-((*S*)-1,1,1-trifluoropropan-2-yl)acetamide (9)**

The title compound **(9)** was prepared in 72% yield from **(7)** in analogous fashion.

^1^H NMR (DMSO-d_6_, 120 °C) δ: 8.57 (1H, s), 8.23 (1H, d, *J*= 0.6), 8.00 (1H, dd, *J* = 8.0, 1.7), 7.96 (1H, d, *J* = 0.7), 7.87 (1H, m), 7.69 (1H, dd, *J* = 8.1, 0.7), 7.54 (1H, br s), 7.40 – 7.33 (2H, m), 7.14 (2H, t, *J* = 8.7), 5.28 – 5.14 (1H, m), 4.84 (1H, d, *J* = 17.7), 4.80 (2H, s), 4.64 (1H, d, *J*= 17.7), 4.52 (1H, d, *J*= 17.0), 4.32 (1H, d, *J* = 17.0), 3.04 (2H, dd, *J* = 37.0, 17.9), 2.69 (3H, d, *J* = 4.7), 1.38 (3H, d, *J* = 7.1).

m/z (ES^+^): 615.3 [M+H^+^]^+^

***N*-(4-Fluorobenzyl)-2-((3'*S*,4*S*)-3'-hydroxy-5'-(1-(2-(methylamino)-2-oxoethyl)-1*H*-pyrazol-4-yl)-2,5-dioxo-2',3'-dihydrospiro[imidazolidine-4,1'-inden]-1-yl)-*N*-((*S*)-1,1,1-trifluoropropan-2-yl)acetamide (10)**

To a stirred solution of *N*-(4-fluorobenzyl)-2-((*S*)-5'-(1-(2-(methylamino)-2-oxoethyl)-1H-pyrazol-4-yl)-2,3',5-trioxo-2',3'-dihydrospiro[imidazolidine-4,1'-inden]-1-yl)-*N*-((*S*)-1,1,1-trifluoropropan-2-yl)acetamide **(8)** (2.50 g, 4.07 mmol) in THF (83 mL) and MeOH (83 mL) at 0 °C was added sodium borohydride (0.92 g, 24.29 mmol) in a portionwise fashion, and upon completion of the addition, the reaction mixture was stirred at 0 °C for 30 minutes. The reaction mixture was quenched by dropwise addition of acetone (4 mL) and concentrated under reduced pressure. Purification by flash column chromatography, eluting with 5% (MeOH + 5% NH_4_OH)/DCM afforded the title compound **(10)** as an off-white solid (1.86 g, 74%).

^1^H NMR (DMSO-d_6_, 120 °C) δ: 8.38 (1H, s), 8.08 (1H d, *J* = 0.7), 7.83 (1H, d, *J* = 0.7), 7.59 (1H, m), 7.52 (2H, dd, *J* = 7.9, 1.7), 7.41 – 7.35 (2H, m), 7.29 (1H, d, *J* = 8.0), 7.15 (2H, t, *J* = 9.0), 5.35 (1H, q, *J* = 6.5), 5.22 (1H, m), 4.95 (1H, d, *J* = 7.0), 4.84 (1H, d, *J* = 17.7), 4.80 (2H, s), 4.64 (1H, d, *J* = 17.7), 4.52 (1H, d, *J* = 17.0), 4.32 (1H, d, *J* = 17.0), 2.69 (3H, d, *J* = 4.8), 2.49 (2H, ddd, *J* = 53.4, 13.3, 6.8), 1.39 (3H, d, *J* = 7.0).

m/z (ES^+^): 617.3 [M+H^+^]^+^

***N*-(4-Fluorobenzyl)-2-((3'*R*,4*R*)-3'-hydroxy-5'-(1-(2-(methylamino)-2-oxoethyl)-1*H*-pyrazol-4-yl)-2,5-dioxo-2',3'-dihydrospiro[imidazolidine-4,1'-inden]-1-yl)-*N*-((*S*)-1,1,1-trifluoropropan-2-yl)acetamide (11)**

The title compound **(11)** was prepared in 93% yield from **(9)** in analogous fashion.

^1^H NMR (DMSO-d_6_, 120 °C) δ: 8.38 (1H, s), 8.08 (1H d, *J* = 0.7), 7.83 (1H, d, *J* = 0.7), 7.59 (1H, m), 7.52 (2H, dd, *J* = 7.9, 1.7), 7.41 – 7.35 (2H, m), 7.29 (1H, d, *J* = 8.0), 7.15 (2H, t, *J* = 9.0), 5.35 (1H, q, *J* = 6.5), 5.22 (1H, m), 4.95 (1H, d, *J* = 7.0), 4.84 (1H, d, *J* = 17.7), 4.80 (2H, s), 4.64 (1H, d, *J* = 17.7), 4.52 (1H, d, *J* = 17.0), 4.32 (1H, d, *J* = 17.0), 2.69 (3H, d, *J* = 4.8), 2.49 (2H, ddd, *J* = 53.4, 13.3, 6.8), 1.39 (3H, d, *J* = 7.0).

m/z (ES^+^): 617.3 [M+H^+^]^+^

**2-((3'*R*,4*S*)-3'-Fluoro-5'-(1-(2-(methylamino)-2-oxoethyl)-1*H*-pyrazol-4-yl)-2,5-dioxo-2',3'-dihydrospiro[imidazolidine-4,1'-inden]-1-yl)-*N*-(4-fluorobenzyl)-*N*-((*S*)-1,1,1-trifluoropropan-2-yl)acetamide (12, iP300w)**

To a stirred solution of *N*-(4-fluorobenzyl)-2-((3'*S*,4*S*)-3'-hydroxy-5'-(1-(2-(methylamino)-2-oxoethyl)-1H-pyrazol-4-yl)-2,5-dioxo-2',3'-dihydrospiro[imidazolidine-4,1'-inden]-1-yl)-*N*-((*S*)-1,1,1-trifluoropropan-2-yl)acetamide **(10)** (1.50 g, 2.43 mmol) in DCM (100 mL) at -70 °C under an atmosphere of nitrogen was added (diethylamino)sulfur trifluoride (0.59 g, 3.65 mmol) in a dropwise manner. After stirring at -70 °C for 30 minutes, the solution was allowed to warm to -30 °C over 10 minutes before being quenched by addition of calcium carbonate (0.75 g). The reaction mixture was allowed to warm to ambient temperature and was then concentrated under reduced pressure. Purification by flash column chromatography, eluting with 3.5% (MeOH + 5% NH_4_OH)/DCM, afforded a white solid. Trituration with diethyl ether followed by lyophilisation from MeCN/H_2_O (1:1) afforded the title compound **(12, iP300w)** as a white solid (0.79 g, 53%).

^1^H NMR (DMSO-d_6_, 120 °C) δ: 8.63 (1H, br s), 8.14 (1H, d, *J*= 0.6), 7.89 (1H, d, *J*= 0.6), 7.73-7.65 (2H, m), 7.51 (1H, br s), 7.38-7.31 (3H, m), 7.13 (2H, t, *J*= 8.8), 6.15 (1H, ddd, *J*= 57.6, 6.8, 4.6), 5.23-5.15 (1H, m), 4.82 (1H, d *J*= 17.6), 4.78 (2H, s), 4.61 (1H, d, *J*= 17.6), 4.48 (1H, d, *J*= 16.8), 4.27 (1H, d, *J*= 16.8), 3.08 (1H, ddd, *J*= 14.4, 12.4, 6.8), 2.68 (3H, d, *J*= 4.4), 2.42 (1H, ddd, *J*= 25.5, 14.4, 4.6), 1.38 (3H, d, *J*= 7.2).

^13^C NMR (DMSO-d_6_, 100 °C) δ: 174.33 (d, *J*_CF_ = 4 Hz), 167.38, 166.24, 160.91  (d, *J*_CF_ = 4 Hz), 154.88, 140.97 (d, *J*_CF_ = 19 Hz), 138.00 (d, *J*_CF_ = 5 Hz), 136.38, 134.09 (d, *J*_CF_ = 3 Hz), 133.01, 128.35, 127.76, 127.68, 127.14 (d, *J*_CF_ = 3 Hz), 125.07 (q, *J*_CF_ = 284 Hz), 123.43, 121.03, 114.64 (d, *J*_CF_ = 22 Hz), 91.87 (d, *J*_CF_ = 177 Hz), 68.25 (d, *J*_CF_ = 1.5 Hz), 54.08,  50.90, 45.19, 42.94 (d, *J*_CF_ = 20 Hz), 39.9, 25.03, 11.05.

^19^F NMR (DMSO-d_6_, 120 °C): -72.09, -115.92, -159.80.

HRMS (ES+) calculated for [C_29_H_27_F_5_N_6_O_4_+H^+^]^+^ found: 619.2079.

[α]_D_^24^ = -41.8° (c = 1, MeOH).

**2-((3'*S*,4*S*)-3'-Fluoro-5'-(1-(2-(methylamino)-2-oxoethyl)-1*H*-pyrazol-4-yl)-2,5-dioxo-2',3'-dihydrospiro[imidazolidine-4,1'-inden]-1-yl)-*N*-(4-fluorobenzyl)-*N*-((*S*)-1,1,1-trifluoropropan-2-yl)acetamide (13)**

The *(S, S, S)-*diastereomer **(13)** was isolated by flash column chromatography as the minor reaction side-product alongside **(12)**.

^1^H NMR (DMSO-d_6_, 120 °C) δ: 8.34 (1H, br s), 8.13 (1H, d, *J*= 0.8), 7.88 (1H, d, *J*= 0.8), 7.71 (1H, m), 7.68 (1H, dt, *J*= 8.0, 1.8), 7.52 (1H, br s), 7.39-7.35 (3H, m), 7.14 (2H, t, *J*= 8.8), 6.23 (1H, ddd, *J*= 57.6, 6.2, 3.8), 5.24-5.16 (1H, m), 4.84 (1H, d, *J*= 17.6), 4.78 (2H, s), 4.63 (1H, d, *J*= 17.6), 4.50 (1H, d, *J*= 16.6), 4.29 (1H, d, *J*= 16.6), 2.76-2.73 (1H, m), 2.71-2.64 (4H, m), 1.38 (3H, d, *J*= 6.8).

m/z (ES^+^): 619.3 [M+H^+^]^+^

[α]_D_^24^ = -33.6° (c = 1, MeOH).

**2-((3'*S*,4*R*)-3'-Fluoro-5'-(1-(2-(methylamino)-2-oxoethyl)-1*H*-pyrazol-4-yl)-2,5-dioxo-2',3'-dihydrospiro[imidazolidine-4,1'-inden]-1-yl)-*N*-(4-fluorobenzyl)-*N*-((*S*)-1,1,1-trifluoropropan-2-yl)acetamide (14, iP300v)**

The title compound **(14, iP300v)** was prepared in 61% yield from **(11)** in analogous fashion.

^1^H NMR (DMSO-d_6_, 120 °C) δ: 8.63 (1H, br s), 8.14 (1H, d, *J*= 0.6), 7.89 (1H, d, *J*= 0.6), 7.73-7.65 (2H, m), 7.51 (1H, br s), 7.38-7.31 (3H, m), 7.13 (2H, t, *J*= 8.8), 6.15 (1H, ddd, *J*= 57.6, 6.8, 4.6), 5.23-5.15 (1H, m), 4.82 (1H, d *J*= 17.6), 4.78 (2H, s), 4.61 (1H, d, *J*= 17.6), 4.48 (1H, d, *J*= 16.8), 4.27 (1H, d, *J*= 16.8), 3.08 (1H, ddd, *J*= 14.4, 12.4, 6.8), 2.68 (3H, d, *J*= 4.4), 2.42 (1H, ddd, *J*= 25.5, 14.4, 4.6), 1.38 (3H, d, *J*= 7.2).

m/z (ES^+^): 619.3 [M+H^+^]^+^

[α]_D_^24^ = +20.8° (c = 1, MeOH).
