# Supplementary material for: Inactivation of the CIC-DUX4 oncogene through P300/CBP inhibition, a therapeutic approach for CIC-DUX4 sarcoma
Source: Oncogenesis. 2021 Oct 12;10(10):68. doi: 10.1038/s41389-021-00357-4 (PMC8511258; doi:10.1038/s41389-021-00357-4)
Supplement: Supplementary file 13 — Supplementary western blots [file 41389_2021_357_MOESM13_ESM.pptx]

## Slide 1
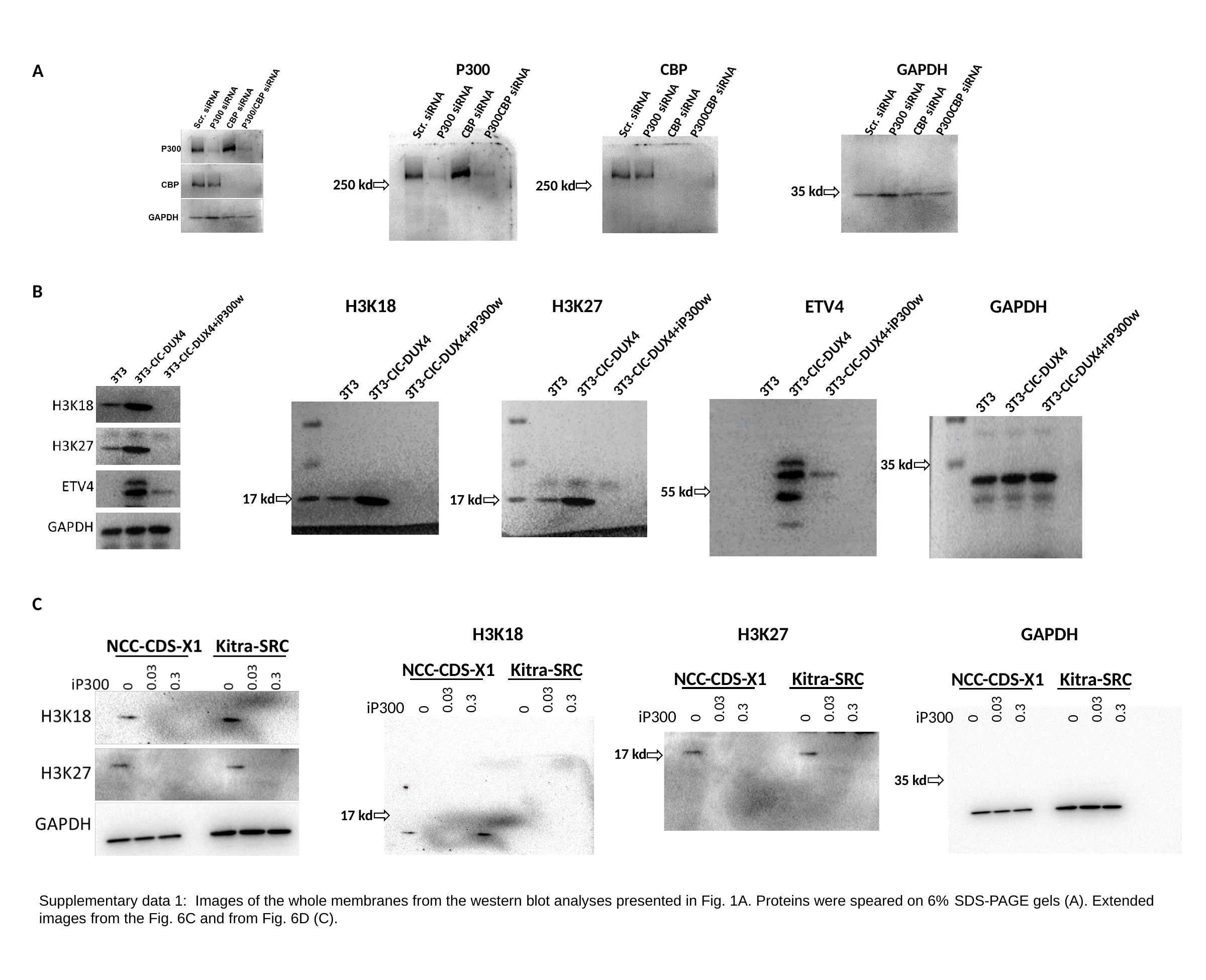

P300CBP siRNA
P300 siRNA
CBP siRNA
Scr. siRNA
P300
CBP
GAPDH
A
P300CBP siRNA
P300CBP siRNA
P300 siRNA
CBP siRNA
P300 siRNA
CBP siRNA
Scr. siRNA
Scr. siRNA
250 kd
250 kd
35 kd
H3K27
3T3-CIC-DUX4+iP300w
3T3-CIC-DUX4
3T3
 17 kd
ETV4
3T3-CIC-DUX4+iP300w
3T3-CIC-DUX4
3T3
 55 kd
H3K18
3T3-CIC-DUX4+iP300w
3T3-CIC-DUX4
3T3
 17 kd
B
GAPDH
3T3-CIC-DUX4+iP300w
3T3-CIC-DUX4
3T3
 35 kd
H3K18
H3K27
GAPDH
NCC-CDS-X1
Kitra-SRC
NCC-CDS-X1
Kitra-SRC
NCC-CDS-X1
Kitra-SRC
0.03
0.03
0.3
0.3
iP300
0
0
 35 kd
0.03
0.03
0.3
0.3
iP300
0.03
0.03
0
0
0.3
0.3
iP300
0
0
 17 kd
 17 kd
C
Supplementary data 1: Images of the whole membranes from the western blot analyses presented in Fig. 1A. Proteins were speared on 6% SDS-PAGE gels (A). Extended images from the Fig. 6C and from Fig. 6D (C).
